# Unsupervised detection of building destruction during war from publicly available radar satellite imagery

**DOI:** 10.1093/pnasnexus/pgaf367

**Published:** 2025-12-09

**Authors:** Daniel Racek, Qi Zhang, Paul Wilhelm Thurner, Xiao Xiang Zhu, Göran Kauermann

**Affiliations:** Institute of Statistics, Ludwig-Maximilians-University Munich, 80539 Munich, Germany; School of Engineering and Design, Technical University of Munich, 80333 Munich, Germany; Institute of Political Science, Ludwig-Maximilians-University Munich, 80538 Munich, Germany; School of Engineering and Design, Technical University of Munich, 80333 Munich, Germany; Munich Center for Machine Learning, 80538 Munich, Germany; Institute of Statistics, Ludwig-Maximilians-University Munich, 80539 Munich, Germany

**Keywords:** destruction, conflict, remote sensing, satellites, robust statistics

## Abstract

Automated detection of building destruction in conflict zones is crucial for human rights monitoring, humanitarian response, and academic research. However, existing approaches (i) rely on proprietary satellite imagery, both expensive and of limited availability at wartime, (ii) require labeled training data, usually not available in war-affected regions, or (iii) use optical imagery, regularly obstructed by cloud cover. This study addresses these challenges by introducing an unsupervised method to detect destruction at the building level using freely and globally available Sentinel-1 synthetic aperture radar images from the European Space Agency. By statistically assessing interferometric coherence changes over time, unlike existing approaches, our method enables the detection of destruction from a single satellite image, allowing for near real-time destruction assessments every 12 days. We provide a continuous, statistically grounded probability measure for the likelihood of destruction at both the building and pixel level, thereby quantifying the level of uncertainty of the detection. Using ground truth data and reported sequences of events, we validate our approach both quantitatively and qualitatively, across three case studies in Beirut, Mariupol, and Gaza, demonstrating its ability to accurately identify the spatial patterns and timing of destruction events. Using open-access data, our method offers a scalable, global, and cost-effective solution for monitoring building destruction in conflict zones.

Significance Statement Understanding the extent and timing of building destruction during conflicts is crucial for improving crisis response and advancing our understanding of conflicts. Yet, current approaches are often inaccessible due to their reliance on proprietary satellite data or ground truth labels unavailable in conflict zones. Combining remote sensing techniques with robust statistics, our study introduces an unsupervised algorithm that uses freely available Sentinel-1 radar imagery to detect destruction with uncertainty estimates. Tested across three real-world case studies, our method is able to reconstruct the chronology of destruction events. By leveraging open data, we democratize access to critical tools for conflict monitoring and assessment.

## Introduction

The ability to detect and assess damage and destruction of buildings in conflict zones is important for monitoring human rights, facilitating humanitarian aid, guiding reconstruction efforts, and more generally academic research on armed conflict. Traditionally, data on destruction have come from ground reports or manually inspected satellite images, which is often resource-intensive, prone to bias, and limited in scope. This has led to a recent shift towards automated remote sensing solutions, often using machine or deep learning techniques in combination with high-resolution satellite imagery.

Despite substantial progress, existing methods face significant limitations. Many rely on proprietary high-resolution imagery (e.g. 30 cm), which is expensive, difficult to scale, and often not readily available for regions at war (1, 2). Approaches using publicly available medium-resolution images (e.g. 10–20 m), on the other hand, face challenges in reliably identifying the destruction of individual buildings, hence recently introduced techniques rely on multiple images taken over a longer period of time for detection (1, 3, 4). As a result, they cannot provide near real-time assessment of destruction patterns. Another limitation of most methods is that they are supervised (3–6), requiring labeled ground truth data for training, which is, at least initially, unavailable in war-affected regions. Finally, many existing approaches rely on optical satellite images (1, 2, 6), which are frequently obstructed by cloud cover, further limiting the timely detection of destruction.

**Fig. 1.**
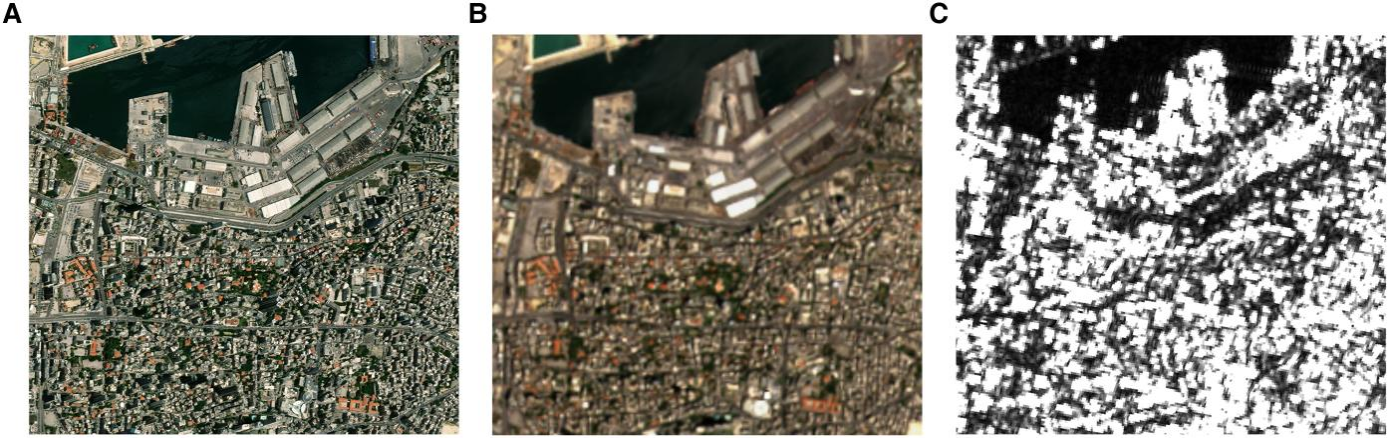
Comparison of satellite images of the Beirut harbor, July 2020. A) A proprietary high-resolution (30 cm) optical image from Maxar WorldView-3. Due to the harbor explosion, the image was made freely available by Maxar (8). B) A publicly available optical image with medium-resolution (10 m) from Sentinel-2A, using bands 4, 3, and 2. C) A publicly available multilooked SAR image with medium-resolution (20 m after processing; see Methods for details) from Sentinel-1. It is one of the images used in our detection. For visualization of the 2D image of complex samples with a real and imaginary part, we use a $\gamma_{0}$ grayscale visualization of the VV polarization.

To address these limitations, in this work, we propose an unsupervised solution to detect building destruction caused by armed conflict and war, using publicly available imagery from the European Space Agency (ESA), specifically synthetic-aperture radar (SAR) images from Sentinel-1, in 12-day time periods, available globally since 2016. Unlike optical satellite imagery, which relies on clear weather and daylight for optimal image quality, SAR technology can operate day and night under all weather conditions (5, 7), making it particularly suitable for conflict zones. Although the spatial resolution of Sentinel-1 imagery is comparably low (20 m after processing; see also Fig. 1), our approach is able to identify destruction every 12 days at the building level.

We employ Interferometric SAR (InSAR) to measure the stability of an area between two SAR images acquired at different points in time (9, 10). We repeatedly calculate these interferometric coherence scores of temporally adjacent images over extended periods of time. Based on a statistical assessment, using nonparametric median regressions and outlier-robust estimation techniques, this allows us to differentiate destruction from random background noise for each time period, one of the central challenges for change detection techniques (11). Generally, in the remote sensing literature, change detection refers to a set of methods that aim to identify changes in the earth’s surface, with broad applications in fields such as urban development (12), agriculture (13), and land cover monitoring (14), using various types of satellite images with varying levels of resolution. For a recent overview of the literature, we refer the reader to (5, 15, 16). For an introduction to SAR imagery and its applications, we refer to (7, 9). In Fig. 1, we provide a comparison of different satellite images, including a Sentinel-1 image.

Our approach is fully unsupervised and hence does not require any labeled training data, making it applicable in scenarios where ground truth data are sparse or even entirely unavailable. This is particularly relevant in the early stages of wars and in conflicts that receive less media attention, where little information on damage and destruction is available (3). Contrary to destruction caused by natural disasters, conflict-related scenarios typically exhibit extreme class imbalance, as destruction is limited to comparatively few buildings (1, 6). Notably, our approach remains robust to this imbalance. Moreover, it provides a continuous, statistically grounded probability measure for the likelihood of destruction at both the building and pixel level. This stands in contrast to most existing approaches, which generally lack a measure of uncertainty (17) and focus exclusively on classifying either pixels or buildings (5).

Focusing on three case studies—Beirut, Mariupol, and Gaza—each with different destruction dynamics, we demonstrate both quantitatively as well as qualitatively that our method can reliably detect the destruction of buildings and also determine the timing of the destruction, using ground truth data and reported sequences of events for validation. Our results highlight the potential of using freely available satellite imagery to detect destroyed buildings during armed conflict and war at scale, as our approach can be transferred to any other place or region in the world.

## Results

### Beirut

The Beirut harbor explosion on 2020 August 4, constitutes our first case study. Although not caused by armed conflict, the explosion’s effects resemble those observed in conflict-related destruction, with extensive damage concentrated within a densely populated area. This event is particularly practical for analyzing our method, as destruction is limited to a single day, providing a clear temporal boundary for our detection. Furthermore, ground truth data for buildings located in the harbor that were fully destroyed by the explosion are available and field-validated.

**Fig. 2.**
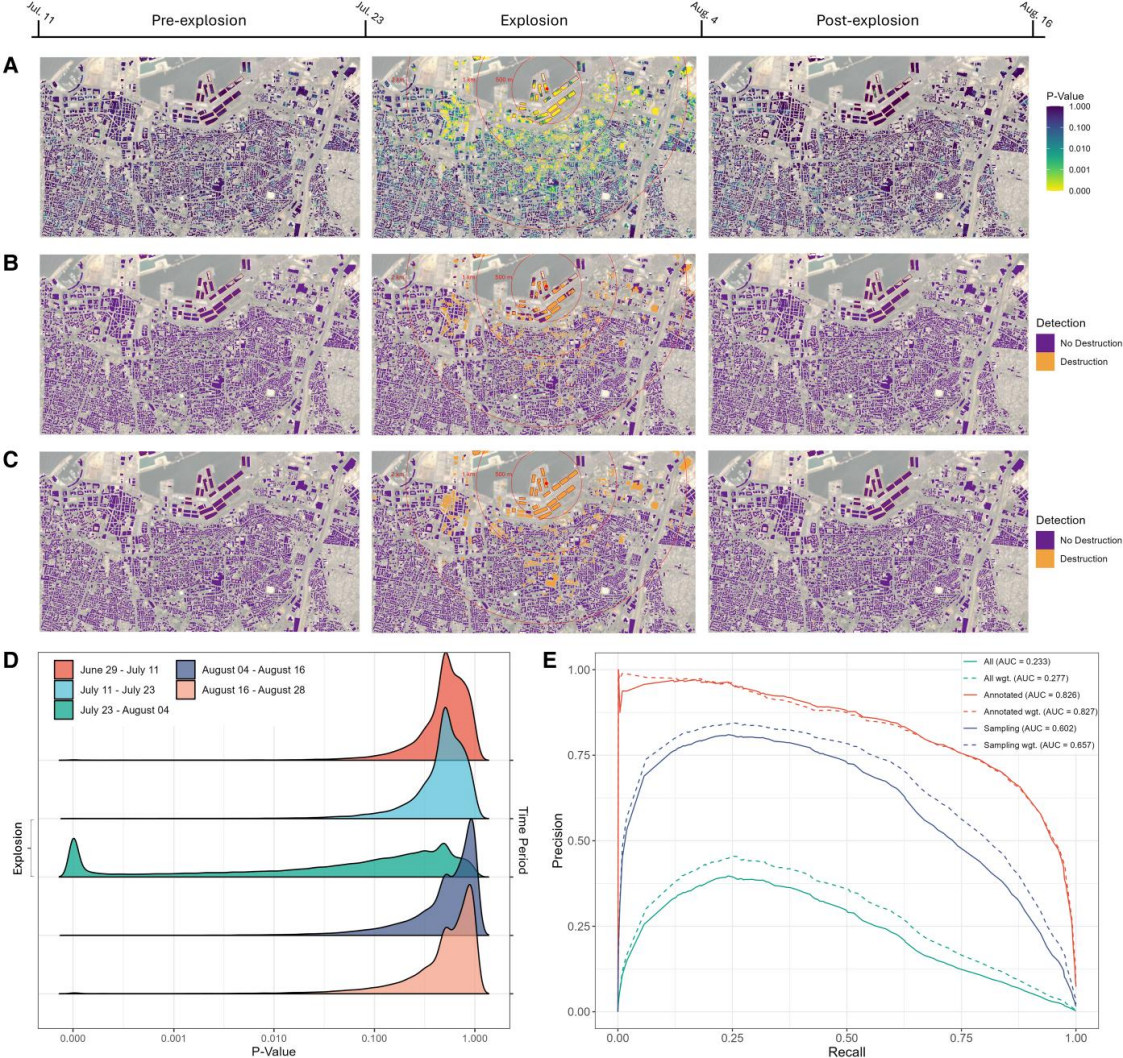
A) *P*-values of destruction of all 10m×10m building pixels in Beirut over 12-day periods from July 11 to July 23 (left), July 23 to August 4 (middle), August 4 to August 16 (right; all 2020). Lower *P*-values indicate a higher likelihood that part of a building was destroyed. The harbor explosion on August 4 is denoted by the red dot in the middle image, with radii of the blast wave with varying distances (also in red). Buildings located directly next to the sea are missing some pixels due to the processing of the images. The background of each image is an optical Sentinel-2A image (freely available) from 2020 July 24 based on bands 4, 3, and 2. B) The same building pixels over the same time periods as in (A), categorized into destruction and no destruction for an F1-score-optimizing probability threshold. C) Classification of entire buildings into destruction and no destruction for an F1-score-optimizing probability threshold after combining pixel-wise *P*-values. D) Kernel density estimates of the distribution of all building pixel *P*-values over 12-day time periods before, during and after the explosion (all 2020). E) Precision-recall curves using the building pixels of the annotated buildings only (red), using random sampling for the negative class (blue), using all building pixels over all time periods (green). The dashed lines denote the curves when pixels are weighted by their building coverage.

Figure 2A visualizes *P*-values of destruction of all 10m×10m building pixels over 12-day time periods in the area around the explosion, using our detection algorithm. The first time period marks the 12 days before the explosion, the second covers the time of the explosion, and the third is directly after the explosion. Lower *P*-values indicate a higher likelihood and more evidence that a building was destroyed. We provide the same maps for all time periods in Supplementary material 1. The location of the explosion is denoted by the red dot. Buildings at the harbor annotated as fully destroyed by the explosion are marked by a red border. As evident from the figure, most of the annotated buildings show high evidence of destruction according to our method in the time period of the explosion. Additionally, almost in a perfect radius around the explosion, we identify additional buildings as destroyed. As we move further away from the explosion site, *P*-values increase, representing likely lower levels of damage to these buildings. These findings are in line with previous research (18, 19), which has identified destruction and substantial damage to buildings farther from the explosion site. In both of the other time periods, as expected, there is limited evidence of destruction (high *P*-values).

Figure 2D provides kernel density estimates of the distribution of *P*-values of all building pixels across different time periods. The explosion is clearly evident from the spike in low *P*-values during that period (density in green). Furthermore, we can observe that our approach almost exclusively assigns low *P*-values for the explosion, and not for any other time period. Note that there is an asymmetric spike in *P*-values in the periods after the explosion due to the use of first differences in our detection.

To evaluate our approach in a strict classification setting, we assign the 1,754 pixels of annotated buildings to our positive destruction class, as for these we have definitive evidence that the corresponding buildings were fully destroyed. However, there are different options on how to define the set of pixels of the negative class, i.e. those that were neither damaged nor destroyed, as ground truth information for the remaining buildings in the explosion time period is unfortunately not available. This is a general problem when designing and evaluating change detection algorithms (11, 16) and exacerbated in conflict scenarios (3, 6), thus highlights one of the advantages of our unsupervised approach, which does not need to be trained.

The simplest option is to use all building pixels from all other time periods as our set of data points in the negative class. However, this results in a total of 994,651 data points and thus in an extremely unbalanced sample (0.16% pos. class), for which performance scores are skewed by individual outliers that naturally occur, e.g. due to building construction works. This imbalance also motivates our subsequent use of precision-recall (PR) curves for evaluation (20). Independent of the definition of our evaluation dataset, the corresponding receiver operating characteristic (ROC) curves exhibit almost perfect areas under the curves (AUCs) of >0.985 (see Supplementary material 5) and thus are not well suited for evaluation. The issue of imbalance in the context of detecting building destruction is extensively discussed in Ref. (6), and thus we refer the reader for more information to the work of these authors. Hence, instead, in Fig. 2E, we visualize the PR curves and the corresponding areas under the curve (AUPRCs) when defining varying probability thresholds as cut-offs for categorizing pixels into destruction vs. no destruction, for various definitions of the evaluation dataset.

Another possibility for evaluation is to only use the pixels of the annotated buildings across all other time periods (32,571 data points; 5.39% pos. class). However, this arguably leads to overoptimistic performance scores (red PR curves). We find that an F1-optimizing probability threshold in this setting to classify many building pixels in other time periods incorrectly as destroyed (see Supplementary material 3). Hence, instead, we opted for randomly sampling building pixels across all other time periods, while retaining the imbalance at a reasonable level (155,100 data points; 1% pos. class). In order to reduce noise, we repeat this process 100 times and average the results. Finally, because many pixels only partially cover buildings, as the resolution of most buildings is more fine-grained than our interferometric coherence scores, we additionally weight each pixel by its relative share of building coverage. The corresponding average PR curve is visualized in Fig. 2E in dashed-blue and has a AUPRC of 0.657 (SD=0.010). The F1-optimizing probability threshold, results in an F1 score of 0.663 (SD=0.004), with an associated precision of 0.694 (SD=0.01) and a recall of 0.634 (SD<0.001).

In Fig. 2B, we visualize the corresponding classification of all building pixels over the same three time periods as in Fig. 2A. We provide the maps for all time periods in Supplementary material 2. As evident, using this threshold, we correctly classify most pixels of the annotated buildings as destroyed. Note, not all pixels will always provide the same level of evidence for destruction (e.g. buildings might only be partially damaged or destroyed). Hence, it is reasonable to classify entire buildings as destroyed, even when only some pixels indicate destruction.

To classify buildings, we combine the pixel-wise *P*-values of each building by constructing the harmonic mean *P*-value (HMP) (21), often used for meta-analyses (22). Equivalently to our pixel-wise classification, we randomly sample buildings from other time periods for evaluation (2,200 data points; 1% pos. class), and repeat this process 100 times. The corresponding PR curve has an AUPRC of 0.905 (SD=0.058). The F1-optimizing probability threshold results in an F1 score of 0.905 (SD=0.032), with an associated precision of 0.861 (SD=0.059) and a recall of 0.955 (SD < 0.001). In Fig. 2C, we again visualize this classification. Only a single building (top right in the middle image) is incorrectly classified as not destroyed, while the amount of false positives in the remaining time periods is highly limited. We provide maps for the remaining time periods in Supplementary material 4, and both ROC and PR curves in Supplementary material 5.

In the explosion time period, we classify a total of 361 buildings as destroyed, which are 5.222% of all buildings in our analysis region. In all other time periods, on average, we incorrectly classify only 11.450 (SD=13.407) as destroyed, a share of 0.166% (SD=0.194) over all buildings. Notably, the latter is very likely to be at least partially driven by reconstruction efforts, which are similarly identified by our detection algorithm, as these constitute changes in a building’s structure. Before the explosion, we classify on average 9.250 (SD=13.551) as destroyed, whereas this increases to 12.916 (SD=13.701) after the explosion. We provide further evidence for this theory in Supplementary material 7. We report results for the other two evaluation strategies in Supplementary material 6.

In Supplementary material 8, we present a sensitivity analysis to evaluate the impact of the number of time periods, that is, satellite images, used for the detection. The results show that destruction can be identified immediately within the time period in which it occurs, with as few as eight images observed prior.

### Mariupol

For our second case study, we investigate building destruction during the course of the Russian invasion of Ukraine. Our area of interest is the center of Mariupol, Zhovtnevyi district, for which UNOSAT has compiled ground truth data for a subset of buildings, manually labeled through high-resolution satellite images from Maxar and not field validated. Unlike in Beirut, destruction in Mariupol unfolded over several weeks, allowing us to test our method’s ability to detect destruction over an extended period of time. Note, due to the limited and partially validated nature of the ground truth data, performance evaluation should be interpreted with caution.

**Fig. 3.**
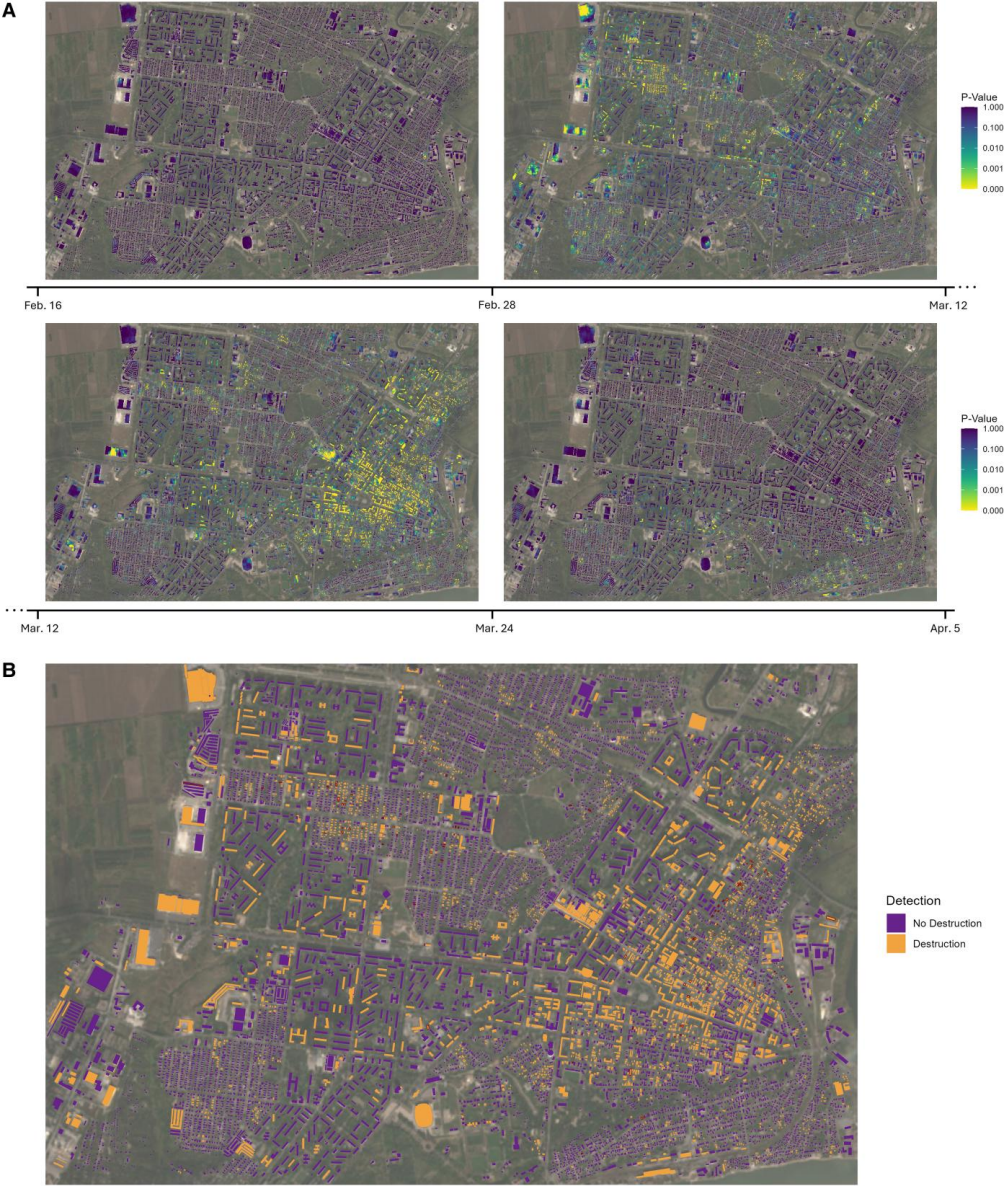
A) *P*-values of destruction of all 10m×10m building pixels in the center of Mariupol (Zhovtnevyi district) over 12-day periods clockwise from February 16 to February 28 (top left), February 28 to March 12 (top right), March 12 to March 24 (bottom left), March 24 to April 5 (bottom right; all 2022). Lower *P*-values indicate a higher likelihood that part of a building was destroyed. The Russian invasion of Ukraine started on 2022 February 24. According to reports, the siege of Mariupol intensified after March 2 (24). The background of each image is an optical Sentinel-2A image (freely available) from 2021 February 10 based on bands 4, 3, and 2. (B) Classification of entire buildings into destruction and no destruction for an F1-score-optimizing probability threshold after combining pixel-wise *P*-values. The classification is summarized across all 12-day time periods from February 28 to May 11, which aligns most closely with the ground truth labels from May 12. On May 16, the fall of Mariupol was officially declared by the Ukrainian army (25). Buildings labeled by UNOSAT as fully destroyed are marked by a red border. Note, this labeling is incomplete. Many more buildings were likely destroyed.

Figure 3A visualizes *P*-values of destruction of all 10m×10m building pixels over 12-day time periods from the start of the invasion to the fall of Mariupol. During the first 4 days of the invasion, the center of Mariupol remained mostly unscathed. Over the course of the following weeks, we can observe how the patterns of destruction move from north-west towards the south-east of the center district, as the Russian army destroys most of the city through bombardments (23). As evident, our approach is capable of tracking these dynamics over the course of the invasion. We provide the same maps for all time periods in Supplementary material 9.

For evaluation, we rely on a limited number of ground truth labels from 2022 May 12. As these only contain buildings that are destroyed, equivalently to the previous application, we sample data points for our negative class by drawing on pixels and buildings for time periods before the invasion. We derive our summarized destruction classification, by categorizing a pixel or building as destroyed when it is marked by our algorithm as destroyed in any of the 12-day time periods from February 28 to May 11.

We report both pixel- and building-level performance scores in Table 1. For each, we present the F1 score, recall, and precision using both the optimal probability threshold derived from the Beirut use case and the F1-maximizing threshold for Mariupol. AUROC and AUPRC are broadly comparable to those observed in the Beirut case study for both pixels and buildings, with the building AUPRC being the notable exception. Unlike in Beirut, where the building AUPRC was exceptionally high, in Mariupol, it is much closer to pixel-level performance. This difference is likely caused by the fact that the large buildings in the Beirut harbor were particularly easy to classify correctly. Although calibration further improves classification performance (+2.06% for pixels and +17.02% for buildings; both F1 scores), our approach performs well overall. For the same reason as previously discussed, building-level calibration also results in a larger performance improvement.

**Table 1.** Mariupol performance

	Pixels	Buildings
Annotations	421	93
AUROC	0.987 (<0.001)	0.992 (<0.001)
AUPRC	0.550 (0.020)	0.650 (0.035)
F1	0.534 (0.011)	0.558 (0.011)
	0.545 (0.014)	0.653 (0.017)
Recall	0.470 (<0.001)	0.430 (<0.001)
	0.587 (<0.001)	0.624 (<0.001)
Precision	0.619 (0.030)	0.795 (0.045)
	0.509 (0.024)	0.687 (0.038)

Performance scores for Mariupol, Zhovtnevyi district, for both pixels and buildings. The annotations denote the number of pixels resp. buildings in the positive destruction class. The data points of the negative class are sampled from periods before the invasion (up to a 99:1 distribution). This process is repeated 100 times. The mean performance scores across these repetitions are reported for each measure with the SD in brackets. For F1, recall, and precision, the first line denotes the corresponding performance score when using the optimal probability threshold from the Beirut case study, the second line denotes the score when using the F1-optimizing threshold.

Based on the optimal classification threshold, we estimate that 2,437 (22.22%) out of 10,964 buildings were completely destroyed in Zhovtnevyi district, with likely many more damaged. We visualize a map for this classification with the corresponding ground truth labels in Fig. 3B. Individual classification maps for each time period are visualized in Supplementary material 10. Both PR and ROC curves are provided in Supplementary material 11.

### Gaza

**Fig. 4.**
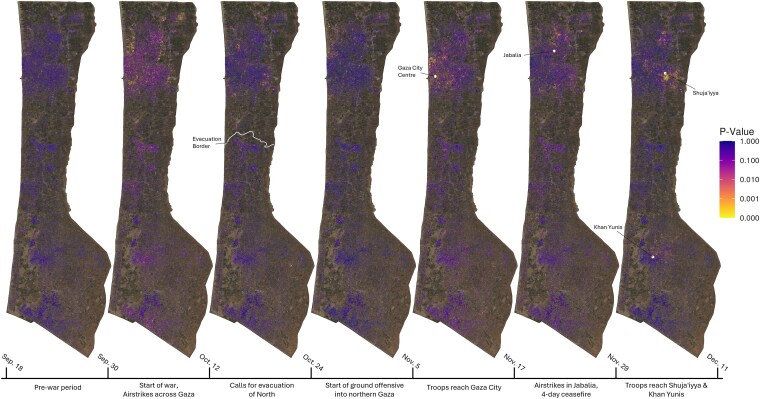
*P*-values of destruction of all 10m×10m building pixels in Gaza over 12-day periods from 2023 September 18 to December 11. Lower *P*-values indicate a higher likelihood that part of a building was destroyed. The background of each image is an optical Sentinel-2A image (publicly available) from 2023 May 5 based on bands 4, 3, and 2. The timeline at the bottom denotes key events taking place between image acquisition dates.

The third case study analyzes the destruction of buildings during the ongoing Israel-Hamas war in Gaza, which began on 2023 October 7. Here, our focus lies on tracking the dynamics of the war over several months. In Fig. 4, we present *P*-values of destruction for building pixels from 2023 September 18 to December 11. Key events are displayed in the bottom time bar. The figure highlights that the dynamics of the war are reflected in patterns of destruction. Following the initial attack on October 7, widespread airstrikes across the Gaza Strip are clearly visible in the second map. Subsequent calls for the evacuation of northern Gaza (north of the drawn evacuation border) correspond to destruction being mostly limited to these areas, as seen in maps 3 and 4. From the fifth map onward, the Israeli ground offensive becomes evident, with destruction closely tracking troop movements, initially in Gaza City (map 5), and later in Shuja’iyya and Khan Yunis (final map). We provide the maps for the remaining time periods in Supplementary material 12.

UNOSAT regularly publishes updated damage statistics for Gaza (see Methods). Using the optimal building classification threshold from our previous case study, we compare their composite destruction estimates with ours in Table 2. Our estimates of the share of destroyed buildings in Gaza follow a trend similar to those of UNOSAT. As expected, due to the unbalanced sampling strategy and a resulting lower classification threshold to reduce false positives, our estimates are more conservative than those of UNOSAT.

**Table 2.** Gaza cumulative building destruction estimates

UNOSAT		Ours	
Date	% destroyed	Date	% destroyed
2023 October 10	0.84	2023 October 12	1.57
2023 November 11	2.99	2023 November 5	2.56
2023 November 26	4.44	2023 November 29	4.34
2024 January 7	10.31	2024 January 4	7.89
2024 February 29	14.25	2024 March 4	10.31
2024 April 1	15.43	2024 March 28	10.69

Comparison of the cumulative share of destroyed buildings estimated by UNOSAT (see Methods) compared to our estimates, based on the optimal classification threshold obtained from Mariupol. Includes all buildings across Gaza over the course of the Israel–Hamas war. The date corresponds to the day of the latest satellite image used for the destruction estimates. As these do not perfectly align due to image acquisition, we use the closest possible match.

In the time periods before the war, we only incorrectly classify on average 0.021% (SD=0.010) buildings as destroyed. We provide corresponding classification maps across all time periods in Supplementary material 13. Additional results from a spatial validation analysis are provided in Supplementary material 19, offering further evidence that our algorithm can reliably detect patterns of destruction during the war in Gaza. A short animation of the sequence of events is available online at link.

## Discussion

In this work, we have presented an unsupervised method for detecting building destruction in conflict zones using freely available Sentinel-1 SAR imagery, and applied it across three case studies, Beirut, Mariupol, and Gaza. We demonstrate that our approach is not only able to identify the destruction of buildings, but, unlike recently introduced techniques (1, 3, 4), also conduct this detection in near real-time. The unsupervised nature of our algorithm eliminates the need for labeled training data, which is often unavailable at the start of wars and conflicts, and thus allows for an application in scenarios where timely information on destruction is of importance.

Our reliance on publicly available satellite data from the ESA makes our method easily accessible and scalable. Sentinel-1 imagery is available globally since 2016 and is completely free of use to both researchers and the public. Although our approach based on lower resolution imagery (20 m) will generally not match the performance of methods using proprietary high-resolution images (e.g. 20 cm), our results demonstrate that it is sufficient to detect patterns of destruction at the building level, without any financial costs for these images. In contrast, acquiring up-to-date high-resolution imagery (20–50 cm) costs ∼$25–50 per km^2^ per image (26, 27). For the entire Gaza Strip, which spans roughly 365 km^2^, a single day of high-resolution coverage would cost between $9,125 and $18,250, with an analysis similar to ours (28 images) costing up to $511,000. It is important to note that high resolution images are not always available for regions at war. Specifically, acquiring these images retrospectively at a high frequency can be difficult or entirely impossible. High-resolution approaches also face challenges in processing the vast amounts of data required for large-scale applications. Our method, on the other hand, is computationally efficient and can be deployed at scale.

An important feature of our algorithm is its use of *P*-values to quantify the evidence for destruction. This statistical framework provides a measure of uncertainty for the detection, and allows users to adjust thresholds based on specific priorities. For example, lower thresholds might be employed for exploratory analyses of broad damage patterns, at the cost of a higher rate of false positives. Beyond threshold adjustment, *P*-values offer users additional flexibility and granularity in their analyses, compared to a strict binary classification. Finally, the use of *P*-values enables a seamless and statistically sound transition between pixel- and building-level analyses, addressing a common limitation in the literature where these approaches are typically treated as mutually exclusive (5).

However, limitations remain. The comparably low resolution of Sentinel-1 imagery prevents the reliable detection of lower levels of damage to buildings, and detection performance will generally be higher for larger structures than for smaller ones. Additionally, while our method performs well without ground truth data, as demonstrated, performance improvements in classification can be achieved through calibration with ground truth labels specific to the corresponding use case. As discussed in detail by Ref. (4), interferometric coherence scores can also be influenced by various other processes unrelated to destruction, including snowfall, the growth and decay of vegetation, floodings, as well as building constructions and repairs. While longer-term shifts, such as those related to seasonality, should be captured through our trend-based methodology, short-term changes like snowfall or building repairs can lead to false positives in the detection, as also seen and discussed in our Beirut results.

Notably, a direct performance comparison with other approaches is challenging because studies use different data samples for evaluation. For example, Ref. (4) analyze building destruction in Ukraine using “stable” pixels (identified in the prewar period) labeled as “urban” or “built-up” by the Global Human Settlement (GHS) BUILT-S dataset. They do not up-sample the negative (no-destruction) class and instead use UNOSAT-labeled pixels from a fixed prewar time window. Ref. (3), who similarly analyze Ukraine, use building information from OpenStreetMap and Microsoft’s Global Building Footprints, with the negative class again defined by UNOSAT-labeled pixels from a fixed (but different) preinvasion time window. For both studies, the exact label distribution is not reported, thus rendering any performance comparison essentially invalid.

Nonetheless, based on our results and experience, we recommend using the proposed algorithm for large-scale, rapid assessments of destruction. In contrast, most other approaches using medium-resolution imagery require multiple images acquired after the destruction event to detect damage, which prevents them from providing results in near real time. Our method is particularly well suited for the early stages of conflicts, when no ground truth labels are available for training supervised approaches. In such cases, it can also be employed to help guide the labeling process. Furthermore, it can serve as a valuable tool for preliminary analyses to inform decisions on where and when to acquire costly high-resolution imagery for more detailed investigations, or in situations where computational resources are scarce. We do not recommend its application for detecting a small number of individually destroyed buildings across a wide area with many buildings, as false positives remain possible. Given the use of an external dataset for building footprints, we see no reason however why the method would not also be applicable in more rural settings.

As also discussed in prior research (6), the automated large-scale detection of destruction during war and conflict allows for the analysis of questions that were previously difficult or impossible to address. For example, they can provide information on when, where, and which regions and types of buildings are targeted and destroyed, including schools, hospitals, and other critical infrastructure. While this supports academic research, such information is particularly valuable for governments and international organizations to help guide humanitarian aid and plan postconflict reconstruction. By offering an objective assessment of destruction, they may also help mitigate potential biases inherent in manually conducted destruction reporting. In the longer term, an interactive online dashboard that continuously updates and visualizes destruction patterns worldwide could further enhance transparency and accessibility, ensuring that information on building destruction is available in near real-time to a broad range of stakeholders. Notably, to prepare such a tool for global deployment, further evaluations across a wider range of contexts, including less densely built environments and varying seasonal conditions, will be important to assess and strengthen its robustness.

## Materials and methods

### Areas of interest

Our areas of interest (AOIs) for analysis of both Beirut and Mariupol are based on available ground truth data (see Destruction labels section). The considered bounding boxes ($x_{\min}$, $x_{\max}$, $y_{\min}$, $y_{\max}$) in the corresponding Universal Transverse Mercator (UTM) zones are for Beirut, UTM zone 36N with a bounding box of 730,447.1, 735,069, 37,51,937, 37,54,567, and for Maripol, UTM zone 37N with a bounding box of 385,635.7, 391,574.4, 52,15,443, and 52,19,254. For Gaza, we consider the whole Gaza Strip, and use UTM zone 36N with a bounding box of 615,957.4, 648,847.4, 34,55,159, and 34,96,499.

### Satellite data

We utilize Sentinel-1 Single Look Complex (SLC) SAR satellite images for our three case studies Beirut, Mariupol, and Gaza. Sentinel-1A and 1B were launched on 2014 April 3 and 2016 April 25, respectively, to a sharing sun-synchronous orbit at 693 km altitude with a repeat cycle of 12 days for a single satellite and 6 days for the two-satellite constellation. The Sentinel-1 satellites each carry a C-band SAR instrument with a central frequency at 5.405 GHz providing acquisitions in all weather and time conditions (28). The SAR sensors aboard can collect images in different operation (Strip Map [SM], Interferometric Wide [IW], Extra Wide [EW], Wave [WV]), and different polarization modes (HH+HV, VH+VV, HH, VV). SAR technology, specifically InSAR, has emerged as a valuable remote sensing tool for damage detection due to its ability to operate under all weather conditions and capture information in both day and night. InSAR coherence, a measure of the stability or similarity between SAR images acquired over the same area at different times, has proven to be a reliable indicator of surface changes (29). High coherence values typically indicate stable and unchanged surfaces, while low coherence values often suggest disruptions or alterations in the structure, such as vegetation growth, ground displacement, or destruction of buildings.

We download Sentinel-1 SLC images for Beirut from 2020 April 6 to December 26 for Mariupol from October 7, 2021 to July 22, 2022, and for Gaza from 2023 May 9 to 2024 April 9, using 12-day intervals for full coverage of each analysis period. The original Sentinel-1 SLC images are captured in IW mode with spatial resolutions of 20 m in the azimuth and 5 m in the ground range direction. These images are then multilooked by a window of 4 pixels in the range direction in order to reduce speckle noise, resulting in Multi-Looked Complex (MLC) images with sampling spacings of 20 m in both azimuth and ground range directions. All downloaded images are freely available from the Copernicus Open Access Hub of the ESA (30).

### Coherence scores

Interferometric coherence is calculated from two coregistered MLC images taken at the beginning and end of each 12-day time period over the same area. The complex images are multiplied pixel-by-pixel to compute coherence, considering both the amplitude and phase information. Specifically, coherence is calculated by averaging the phase differences between the two images over a local window and normalizing it by the product of their amplitudes (9),


(1) $$\gamma =\frac{\left|\sum_{i=1}^{N}S_{1}(i)\cdot S_{2}^{*}(i)\right|}{\sqrt{\sum_{i=1}^{N}|S_{1}(i){|}^{2}\cdot \sum_{i=1}^{N}|S_{2}(i){|}^{2}}},$$

where $S_{1}(i)$ and $S_{2}(i)$ are the complex pixel values from the first and second MLC image, $S_{2}^{*}(i)$ is the complex conjugate of $S_{2}(i)$, and *N* represents the number of pixels in the averaging window. The coherence value, which ranges between 0 and 1, reflects the similarity between the two images: a coherence close to 1 indicates high similarity (minimal change), while a value close to 0 indicates substantial differences, potentially caused by changes in the surface or disturbances.

Decorrelation sources in an interferometric coherence can be represented by a product of different decorrelation components (9),


(2) $$\gamma =\gamma_{\mathrm{SNR}}\cdot \gamma_{\mathrm{temporal}}\cdot \gamma_{\mathrm{spatial}}$$

where, $\gamma_{\mathrm{SNR}}$ is the noise decorrelation due to the signal-to-noise ratio (SNR). $\gamma_{\mathrm{temporal}}$ accounts for temporal decorrelation resulting from changes in the ground surface between the acquisition times of the two MLC images. $\gamma_{\mathrm{spatial}}$ represents spatial or baseline decorrelation depending on the geometry of the satellite passes, particularly the spatial baseline between the two acquisitions. Each decorrelation component takes a value between 0 and 1, with 1 indicating no decorrelation and 0 indicating complete decorrelation. The overall coherence *γ* is thus reduced when any of these decorrelation sources are present.

When buildings or infrastructure are damaged or destroyed between two image acquisitions, the change in surface structure results in a loss of coherence due to temporal decorrelation. In order to detect these temporal changes, SNR and spatial decorrelations are approximated through the use of both digital elevation model (DEM) and satellite trajectories based on InSAR (7, 9, 10), leaving only the temporal decorrelation as the dominant component in the interferometric coherence. The final coherence scores, which are based on sampling spaces of 20 m, are then resampled and projected into UTM, using the corresponding UTM zone of each AOI, to a pixel size of 10m×10m in order to limit the possible loss of information due to the projection.

### Detection of destruction

To reliably identify damage and destruction from changes in the coherence, we analyze the first differences of the coherence values for each pixel over time. For each pixel, we fit a nonparametric median regression with a flexible trend to these differences (31). Median regression is employed to reduce the influence of outliers on the fitted trend, including those resulting from actual destruction. The flexible trend allows us to account for gradual deviations in coherence, such as those caused by atmospheric changes or other nonstructural variations.

We then calculate the differences between observed values and fitted trend, i.e. the residuals. Generally, large residuals can be classified as outliers and are likely due to changes in the building’s structure, such as those caused by damage or destruction. However, since noise levels vary between pixels, this classification is challenging. We solve this by using a robust estimator of the SD, $Q_{n}$, first discussed in Ref. (32).

We find that residual distributions for each pixel to be approximately normally distributed, though with substantially larger tails due to outliers that clearly do not follow the same distribution (see Supplementary material 15). Hence, to estimate the SD of the residuals of each pixel, while being minimally affected by these outliers, we use $Q_{n}$, defined as


(3) $$Q_{n}=d\{|x_{i}-x_{j}|;i<j{\}}_{\left(k\right)},$$

where *d* is a constant and k=(h2), with $h=[\frac{n}{2}]+1$. This means, we take the kth order statistic of all (n2) pairwise distances. The multiplication with *d* is chosen accordingly to achieve consistency. As demonstrated in Ref. (32), this estimator has a low error-sensitivity while simultaneously achieving a high efficiency.

Under the hypothesis of a normal distribution with the estimated SD ${\hat{\sigma }}_{i}$, we can now derive the probability to observe each residual $r_{i,t}$ for a pixel *i* in time period *t*. Specifically, we derive the probability to observe a given residual or a more extreme one in the negative direction, i.e. a one-sided *P*-value with


(4) $$p(r_{i,t})=Pr(R_{i,t}\leq r_{i,t}),$$

where $r_{i,t}$ is the observed residual and $R_{i,t}∼\text{Normal}(0,{\hat{\sigma }}_{i}^{2})$ a random variable. The negative direction is required, as we are only interested in drops in coherence over time.

This *P*-value provides evidence and thus certainty for how likely it is that a given coherence score is not just observed by chance and instead due to structural changes of the building. Higher levels of damage or destruction should lead to lower *P*-values. These *P*-values can either be used directly to visualize likely buildings and areas of destruction, or specific cut-offs can be chosen at which pixels are classified as destroyed.

Using the available ground truth data for Beirut, we experimented with different levels of flexibility in the regression (see Supplementary material 14), different robust scale estimators (see Supplementary material 16), and directly using the coherence scores in the regression instead of their first differences (see Supplementary material 17), before we ultimately decided on the described setup. Note, fitting these median regressions can be carried out at scale, as the computation is straightforward to parallelize. This means even for the Gaza case study, computation only took roughly 2 hours on a single machine. In Supplementary material 8, we additionally conduct a sensitivity analysis to evaluate the impact of the number of available time periods with imagery. The results show that destruction can be identified immediately within the time period in which it occurs, with as few as eight images observed prior.

To classify entire buildings, the *P*-values of each building need to be combined. We do this by constructing the weighted harmonic mean *P*-value (HMP) (21) defined as


(5) $${\bar{\;p}}_{B,t}=\frac{\sum_{\;j=1}^{N}w_{j}}{\sum_{\;j=1}^{N}\frac{w_{j}}{\;p_{\;j,t}}},$$

where *B* refers to the building, *t* to the time period, *N* to the number of pixels that make up the building, $p_{\;j,t}$ the *P*-value of a pixel *j* that is part of building *B*, and $w_{j}$ its corresponding weight based on building coverage.

Compared to alternatives such as Fisher’s method (33) or Stouffer’s Z-score (34), the HMP accounts for the expected positive dependence among the *P*-values of each building, and allows for the incorporation of pixel-level weights based on building coverage. Due to these properties, the HMP is also increasingly adopted in meta-analyses for aggregating *P*-values (22). For an in-depth theoretical discussion with comprehensive simulations and thus motivation for the harmonic mean *P*-value, we refer the reader to Ref. (35).

### Building footprints

To identify individual buildings, we use building footprints from OpenStreetMap (OSM), widely used for applications in urban planning (36), public health (37), and disaster management (38). We obtain these footprints by querying OSM through the *building* key on the first day of the analysis period of each use case, ensuring that each building remains consistent throughout the entire analysis period. This approach prevents any changes, e.g. due to destruction, from being reflected in the building footprints. Using this building, information allows us to only analyze those pixels that constitute buildings. We provide a dataset summary in Table 3.

**Table 3.** Dataset summary

Case study	Pixels	Buildings	No. of time periods
Beirut	57,581	6,915	22
Mariupol	67,737	10,964	24
Gaza	859,502	172,916	28

Number of pixels, buildings, and time periods considered for each case study. The corresponding number of pixels and buildings refers to a single 12-day time period. Each pixel and building is observed over the entire analysis period. Due to the use of first differences in the coherence scores, the first time period drops out.

### Destruction labels

For the Beirut harbor explosion, we utilize georeferenced information on destruction from Ref. (39). The authors draw on ground truth data from the Center for Satellite Based Crisis Information (ZKI) at the German Aerospace Center, who manually annotated buildings based on high-resolution satellite images and field reports. Each labeled building represents a structure at the harbor fully destroyed by the explosion. Note, multiple studies (18, 19) have documented destruction and substantial damage to buildings located farther from the explosion site.

For Mariupol, we use building damage labels in the city center, Zhovtnevyi district, from UNOSAT, part of the United Nations Institute for Training and Research (UNITAR) (40). Selected buildings are annotated manually based on high-resolution (30 cm) optical satellite images from Maxar WorldView-3 on 2022 May 12, with varying levels of visible damage. Although not field-validated, the UNOSAT maps constitute, to the best of our knowledge, the only publicly available, large-scale dataset on building destruction from war and are therefore widely used by journalists, NGOs, and researchers. Consequently, they have been employed as ground truth data in most related studies (1, 3, 4, 6). Due to the missing field validation, we only retain those annotations marked as “high confidence” and labeled as destruction. As each annotation is recorded through a single set of geographic coordinates, we match these to our building footprints to annotate entire buildings.

For the composite damage statistics in Gaza, we similarly utilize information provided by UNOSAT, based on high-resolution (30–50 cm) satellite images from both Maxar and the French national space agency CNES. Theoretically, manual annotations with exact coordinates are available; however, in practice, we find these coordinates to be imprecise. In a vast amount of cases, we cannot successfully match these to our building footprints and hence decided for a composite analysis only. We provide more details in Supplementary material 18.

## Supplementary Material

pgaf367_Supplementary_Data

## Data Availability

All datasets, except SAR imagery, are available at https://osf.io/kw5g9/. Raw satellite images are not provided due to their large file size. However, they are freely available from the Copernicus Open Access Hub of the ESA (30). Additionally, full reproducibility for the remaining parts of the analysis is possible, as all intermediate results and datasets are also provided. Interferometric coherence scores were calculated using Python 3.7 (41) and Gamma (42). The coherence scores were further processed using R 4.3.3 (43). A complete list of all used libraries and their corresponding versions is available in the project’s GitHub repository at https://github.com/Daniel-Rac/Detecting_destruction_from_public_satellite_images. There, we also provide all code needed to reproduce the results.

## Preprints

A preprint of this article is published at https://osf.io/preprints/osf/86t3g.
