# Supplementary material for: Unsupervised detection of building destruction during war from publicly available radar satellite imagery
Source: PNAS Nexus. 2025 Dec 9;4(12):pgaf367. doi: 10.1093/pnasnexus/pgaf367 (PMC12687346; doi:10.1093/pnasnexus/pgaf367)
Supplement: pgaf367_Supplementary_Data [file pgaf367_supplementary_data.pdf]

Supplementary Material

1 Beirut - P-Values

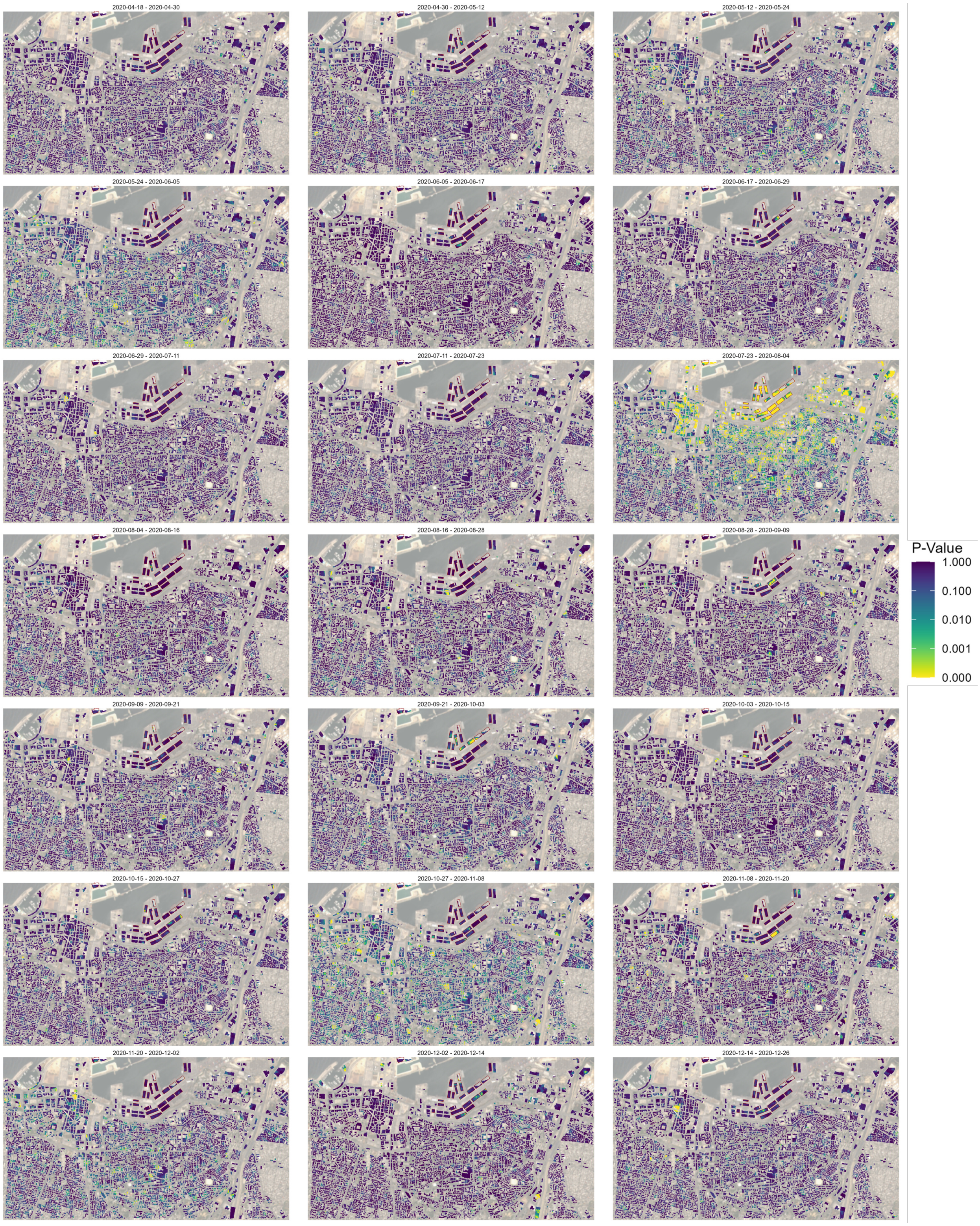

Fig. S1: P-values of destruction of all 10m×10m building pixels in Beirut over all 12-day time periods. Lower p-values indicate a higher likelihood that part of a building was destroyed. The background of each image is an optical Sentinel-2A image (freely available) from July 24, 2020 based on bands 4, 3 and 2.

2 Beirut - Pixel Destruction

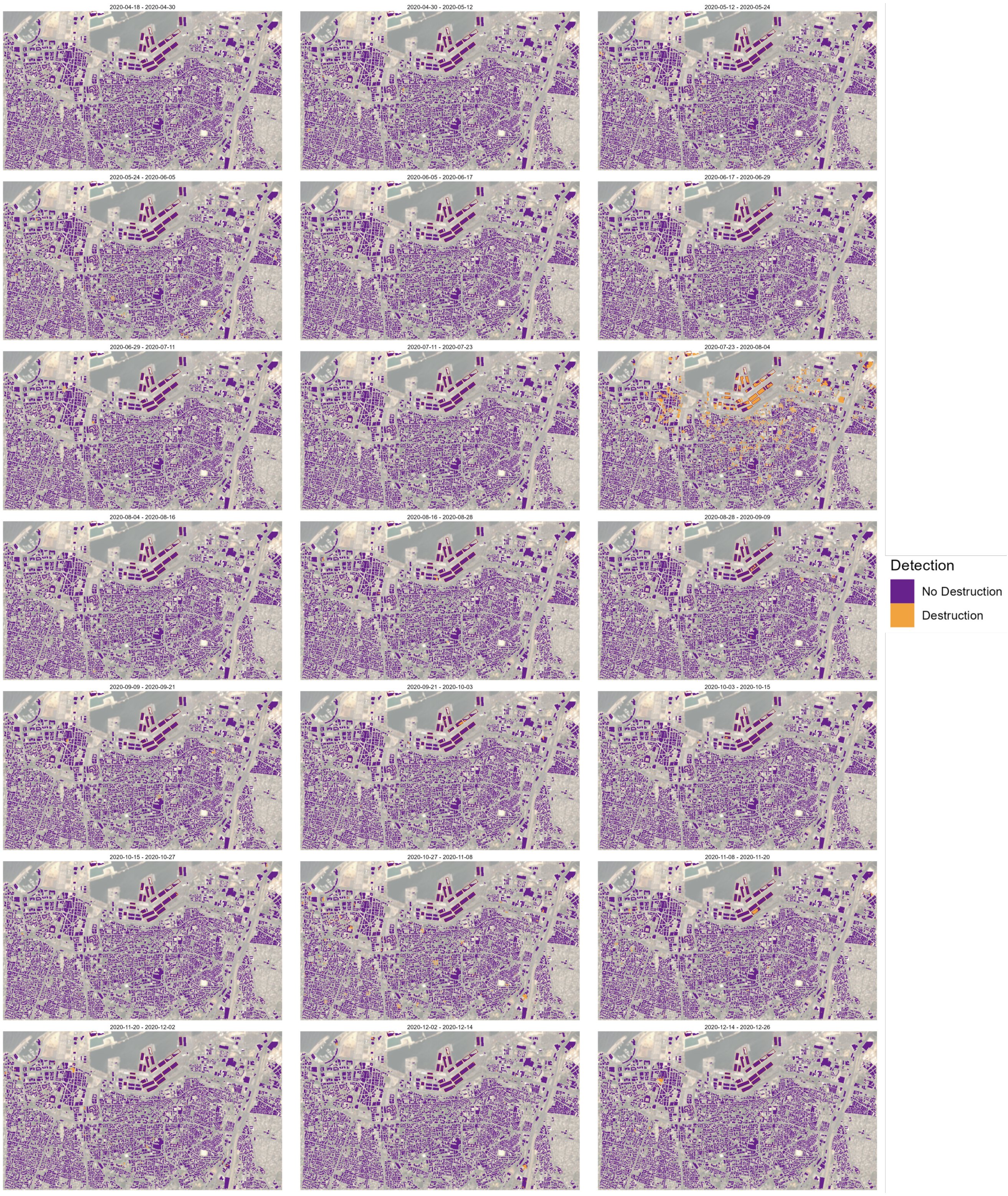

Fig. S2: Classification of all 10m×10m building pixels in Beirut over all 12-day time periods according to the F1-score-optimizing probability threshold, obtained from random sampling. The background of each image is an optical Sentinel-2A image (freely available) from July 24, 2020 based on bands 4, 3 and 2.

3 Beirut - Pixel Destruction - Alternative Threshold

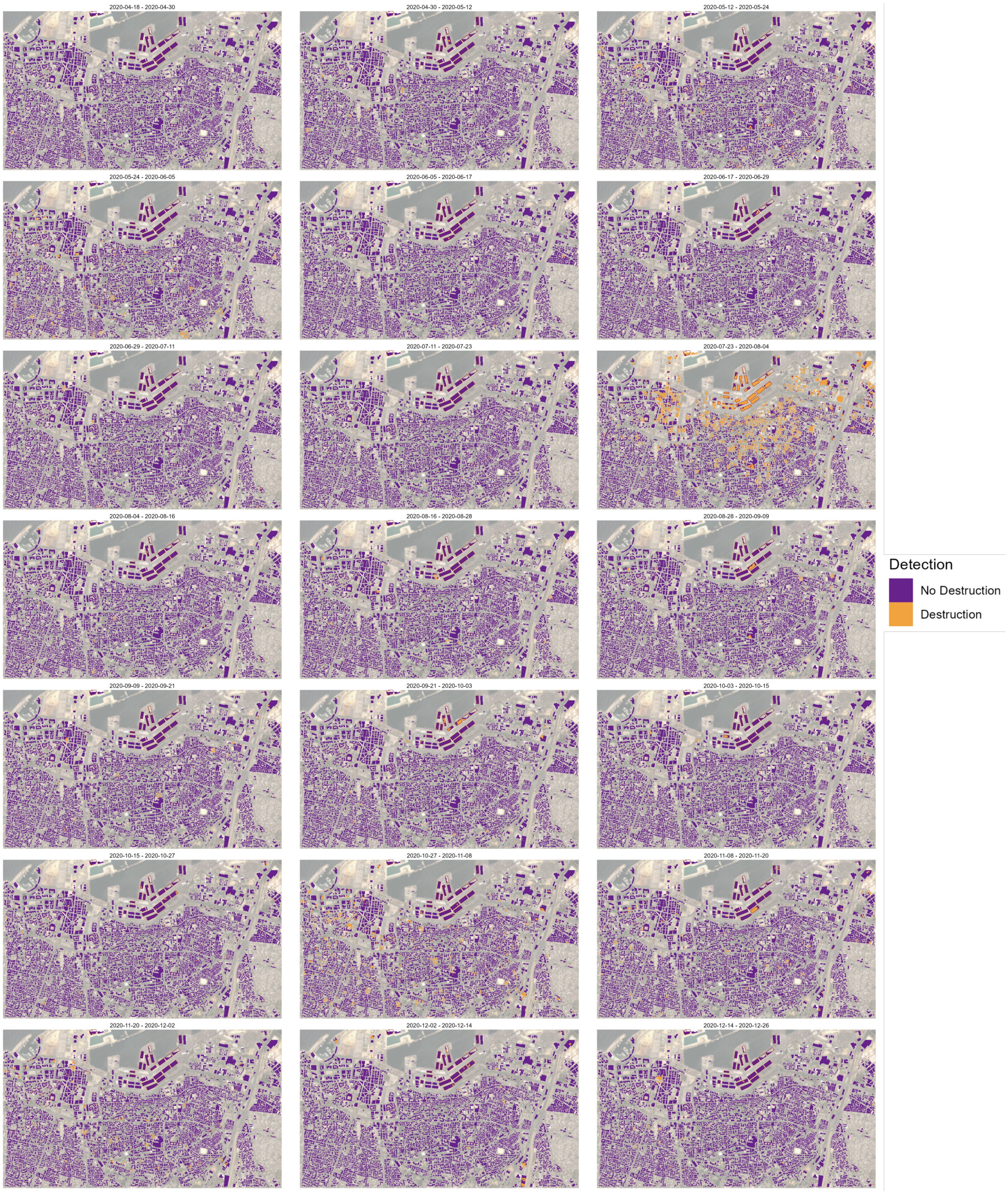

Fig. S3: Classification of all 10m×10m building pixels in Beirut over all 12-day time periods according to the alternative F1-score-optimizing probability threshold, obtained from only using the pixels of the annotated buildings (instead of random sampling). The background of each image is an optical Sentinel-2A image (freely available) from July 24, 2020 based on bands 4, 3 and 2. As evident, the higher probability threshold leads to more false positives across all other time periods.

4 Beirut - Building Destruction

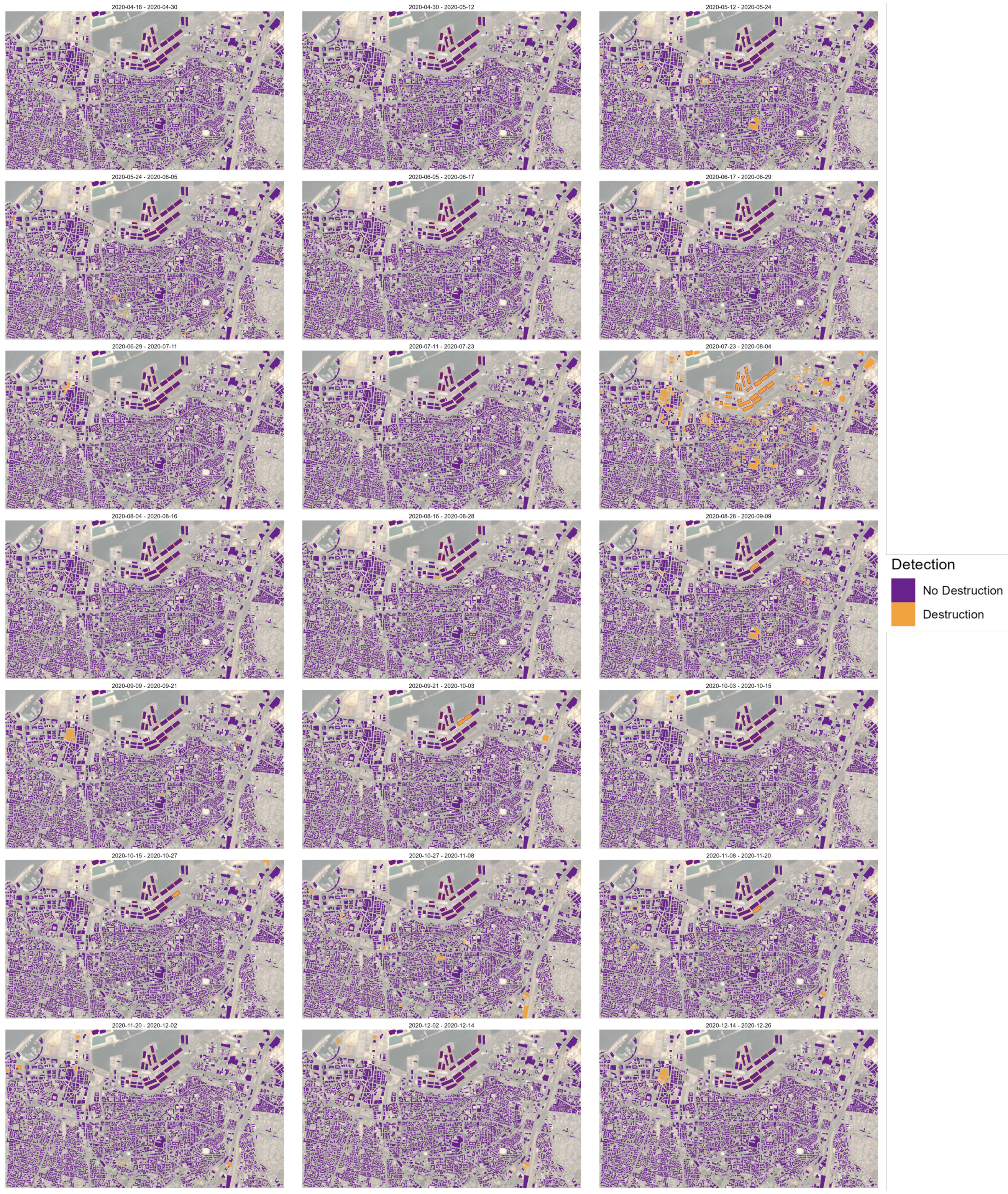

Fig. S4: Classification of entire buildings into destruction and no destruction for an F1-score-optimizing probability threshold, obtained from random sampling, after combining pixel-wise p-values in Beirut. The background of each image is an optical Sentinel-2A image (freely available) from July 24, 2020 based on bands 4, 3 and 2. As evident, the higher probability threshold leads to more false positives across all other time periods.

5 Beirut - ROC & PR Curves

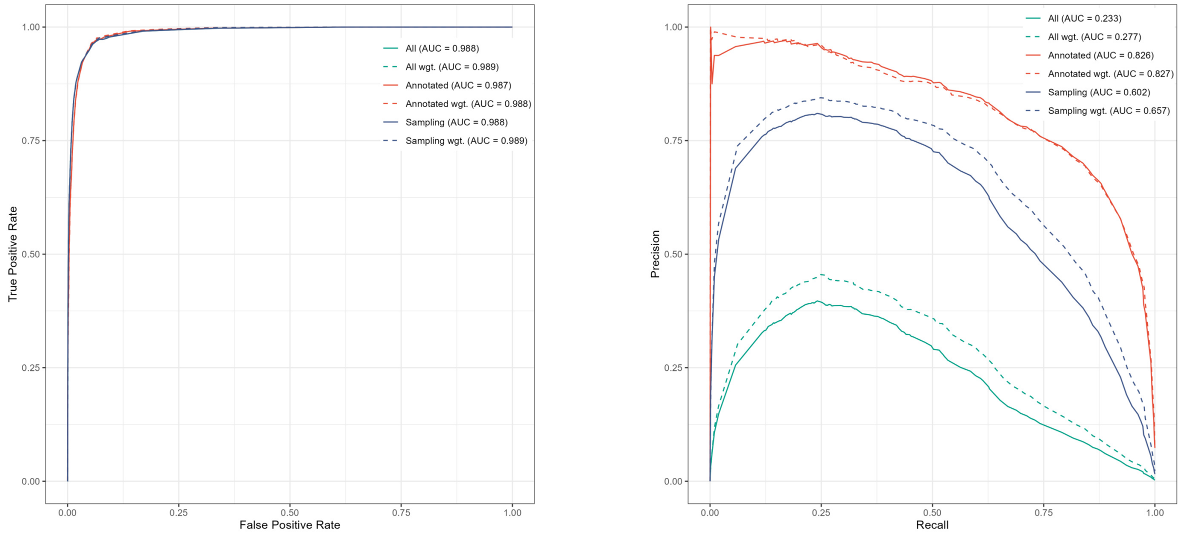

(a) ROC curves **(b)** PR curves

Fig. S5: Performance curves of building pixels for varying evaluation strategies in Beirut.

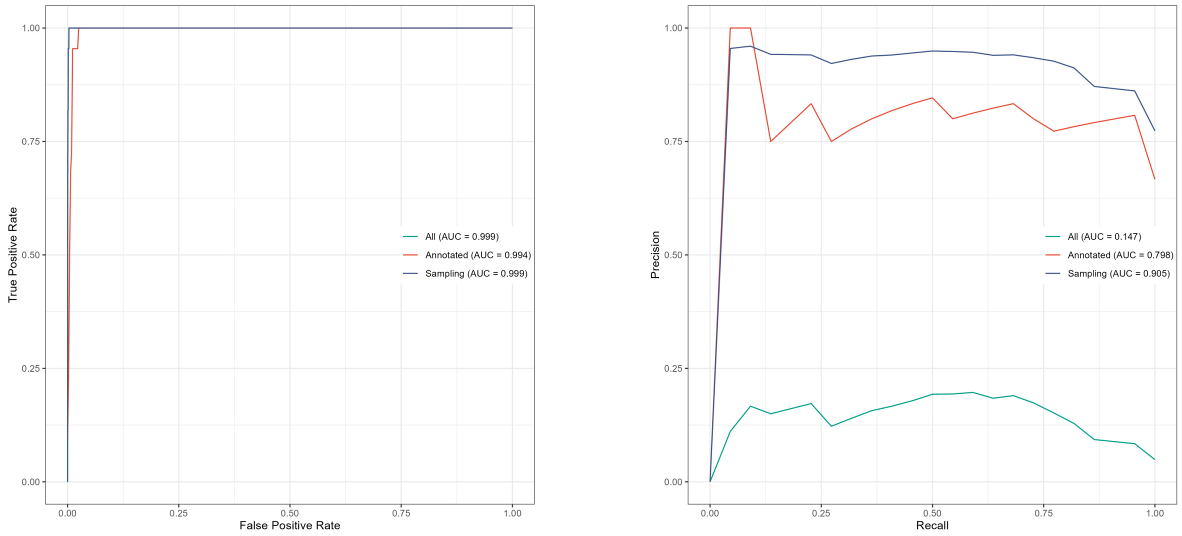

(a) ROC curves **(b)** PR curves

Fig. S6: Performance curves of buildings for varying evaluation strategies in Beirut.

6 Beirut - Performance Results

Table S1: Beirut pixel performance

	All	Annotated	Sampling
**Data points**	94,651	32,571	155,100
**% pos. class**	0.16%	5.39%	1.00%
**AUROC**	0.989	0.988	0.989 (<0.001)
**AUPRC**	0.277	0.827	0.657 (0.010)
**F1**	0.418	0.762	0.663 (0.004)
**Precision**	0.357	0.695	0.694 (0.010)
**Recall**	0.504	0.842	0.634 (<0.001)

Note: Pixel performance scores for Beirut, using all 1,754 annotated building pixels as data points in our positive destruction class. Scores are reported for various definitions of the evaluation dataset. 'All' uses all building pixels across all other time periods (except for the explosion) as the set of data points in the negative class. 'Annotated' only uses the pixels of the annotated buildings across all other time periods. 'Sampling' uses a sampling strategy, for which building pixels of the negative class are sampled from all other time periods (except for the explosion) up to a class distribution of 99:1. This sampling is repeated 100 times and performance scores are averaged across these repetitions, with the standard deviation reported in brackets. For all scores we use their weighted version, where each pixel is weighted by its relative share of building coverage.

Table S2: Beirut building performance

	All	Annotated	Sampling
**Data points**	138,322	462	2,200
**% pos. class**	0.016%	4.76%	1.00%
**AUROC**	0.997	0.990	0.999 (<0.001)
**AUPRC**	0.147	0.798	0.904 (0.058)
**F1**	0.297	0.875	0.905 (0.032)
**Precision**	0.190	0.808	0.861 (0.059)
**Recall**	0.682	0.955	0.955 (<0.001)

Note: Building performance scores for Beirut, using all 22 annotated buildings as data points in our positive destruction class. Scores are reported for various definitions of the evaluation dataset. 'All' uses all buildings across all other time periods (except for the explosion) as the set of data points in the negative class. 'Annotated' only uses the set of annotated buildings across all other time periods. 'Sampling' uses a sampling strategy, for which buildings of the negative class are sampled from all other time periods (except for the explosion) up to a class distribution of 99:1. This sampling is repeated 100 times and performance scores are averaged across these repetitions, with the standard deviation reported in brackets.

7 Beirut - Reconstruction

In the following, we take a closer look at buildings classified as destroyed by our algorithm in the time periods before and after the harbor explosion. As reported in the main manuscript, before the explosion, we classify on average 9.250 (SD = 13.551) buildings as destroyed. After the explosion, this increases to 12.916 (SD = 13.701), i.e., by roughly 40%. We suspect that reconstruction efforts are responsible for this increase, which would be similarly identified by our detection algorithm, as these constitute changes in a building's structure.

In Fig. S7, we visualize density plots of buildings classified as destroyed by our algorithm, based on their distance from the explosion site, for the periods before, during, and after the explosion. The distribution after the explosion is evidently bimodal. The second mode (at higher distances) resembles the pre-explosion distribution, while the first mode (at lower distances) aligns more closely with the explosion distribution. The emergence of the latter mode suggests that some of the destroyed buildings were repaired and/or rebuilt in the months following the explosion, and that these reconstruction efforts are identified by our detection algorithm due to the structural changes they entail. This indicates that our reported performance scores for Beirut are likely conservative, as the evaluation dataset also includes both buildings and pixels after the explosion.

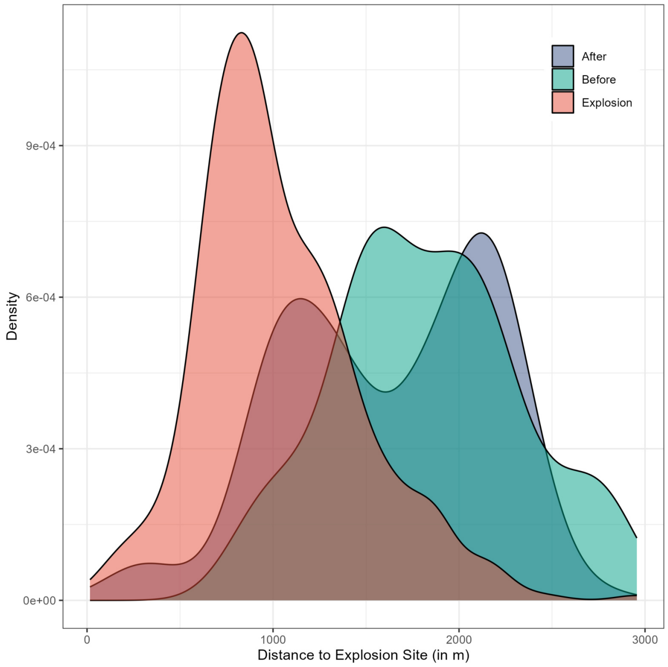

Fig. S7: Density of buildings classified as destroyed by our algorithm by distance to the explosion site, before, during and after the explosion.

8 Beirut - Sensitivity Analysis

Here, we present the results of our sensitivity analysis, evaluating the impact of the number of time periods—and thus the number of satellite images—used for our detection. In Fig. S8a, we show the AUPRC as the number of time periods is gradually reduced, both before and after the explosion period. The full dataset utilizes interferometric coherence scores from 22 time periods (corresponding to 23 satellite images) in total. For the sensitivity analysis, we first remove the last three time periods, reducing the dataset to 9 time periods before and after the explosion (19 in total). We then incrementally remove one time period at both the beginning and end, evaluating performance at each step.

The results show that performance remains stable until the dataset is reduced to nine time periods, at which point both building and pixel-level performance begin to decline. With seven time periods only, performance drops substantially. The observed fluctuations across the steps can be attributed to variations in noise levels across time periods, as well as the influence of reconstruction efforts (see S.6).

In Fig. S8b, we report the AUPRC as the number of time periods after the explosion is gradually reduced. This analysis allows us to evaluate how quickly our algorithm can detect destruction after it occurs. For this evaluation, we consistently retain all 9 time periods before the explosion in the dataset. Based on our previous analysis (Fig. S8a), the general reduction in the number of time periods should only minimally impact these results. The figure shows that the AUPRC remains largely stable throughout. A performance drop is observed only when no time periods after the explosion are included, though it still remains at a reasonable level (-14.3% in pixel performance compared to using all 12 time periods after the explosion; -25.1% in building performance).

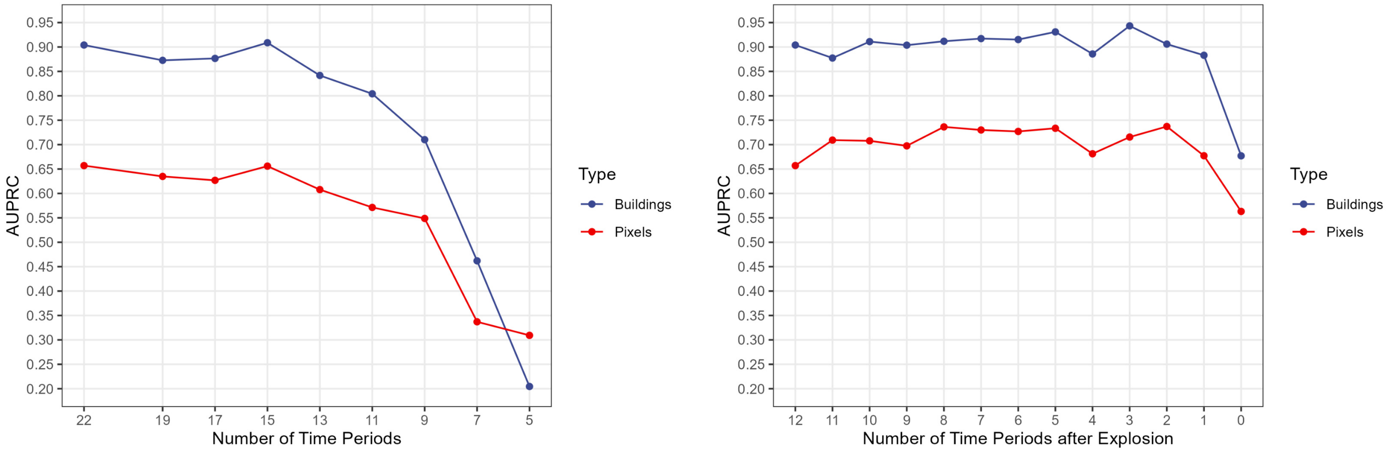

(a) General effect of time periods.

(b) Effect of time periods after the explosion.

Fig. S8: Sensitivity analysis of the effect of the number of time periods on the AUPRC.

9 Mariupol - P-Values

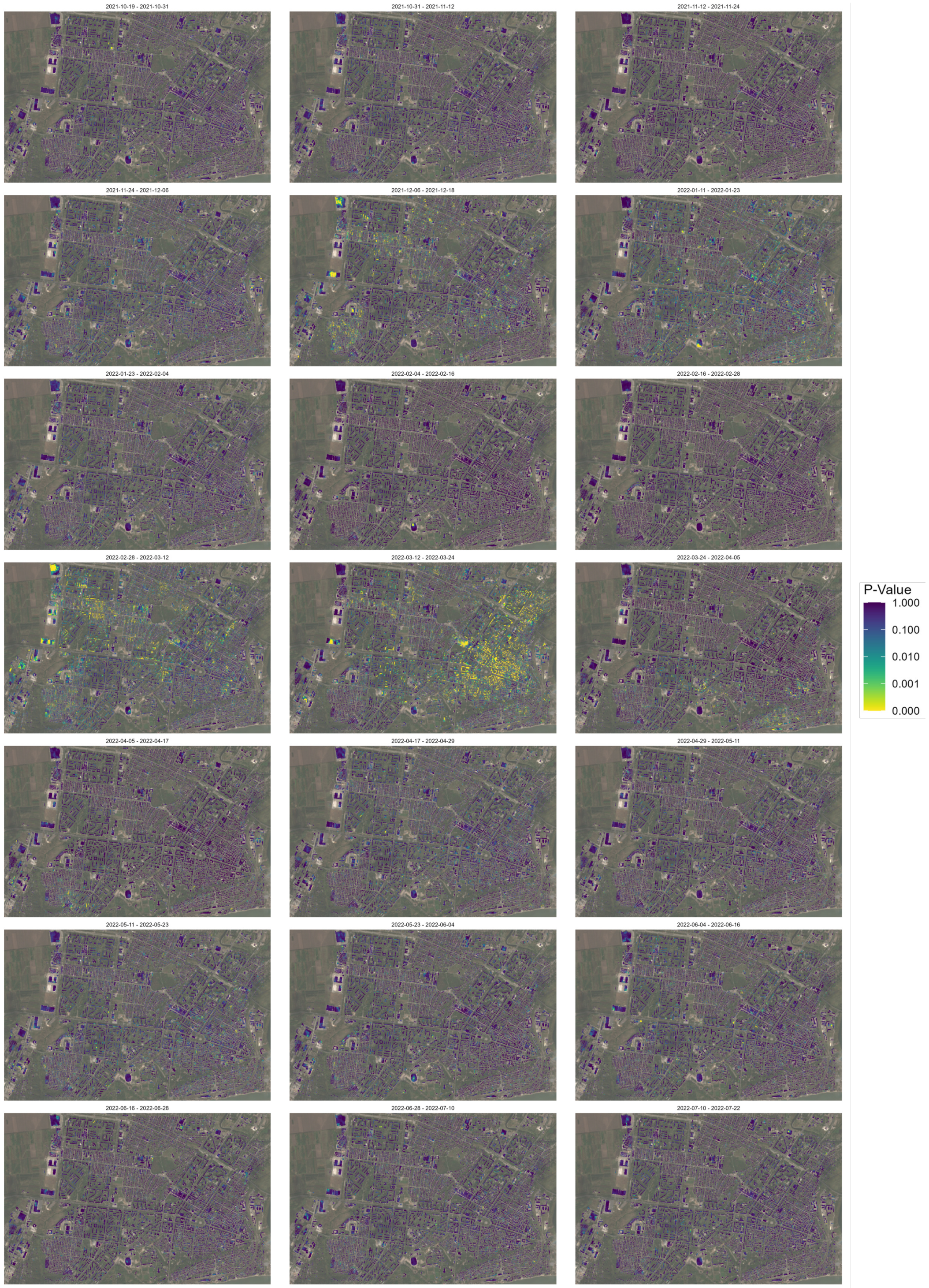

Fig. S9: P-values of destruction of all 10m×10m building pixels in the center of Mariupol (Zhovtnevyi district) over all 12-day time period Lower p-values indicate a higher likelihood that part of a building was destroyed. The Russian invasion of Ukraine started on February 24, 2022. According to reports, the siege of Mariupol intensified after March 2 [Gunter, 2022]. As the image on January 11, 2022 is unfortunately faulty, we had to remove the two 12-day time periods associated with the image from the analysis. The time periods directly before and after are evidently affected by this removal, as pixels generally exhibit lower p-values. Nonetheless, the detection remains mostly stable. The background of each image is an optical Sentinel-2A image (freely available) February 10, 2021 based on bands 4, 3 and 2.

10 Mariupol - Building Destruction

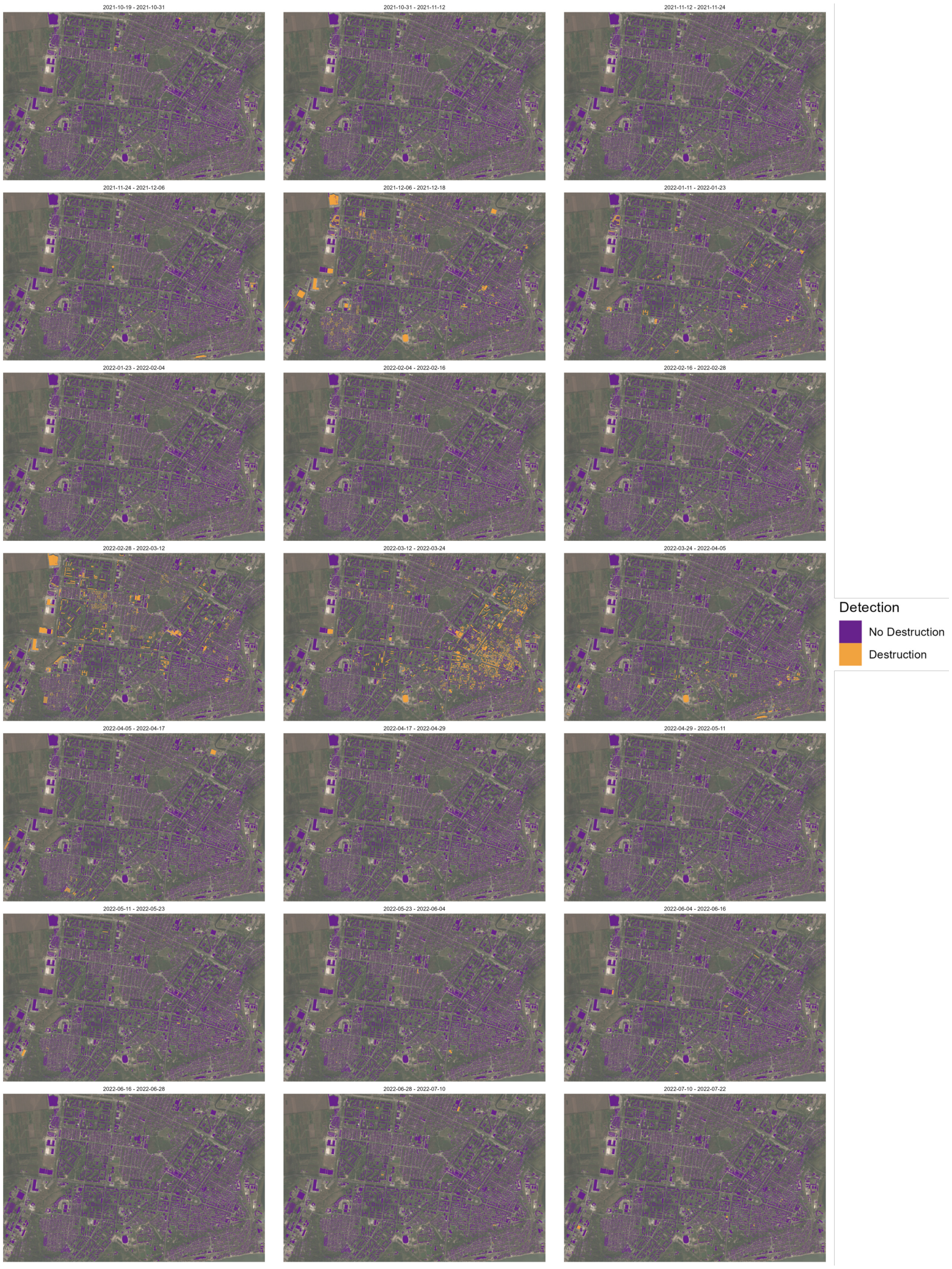

Fig. S10: Classification of entire buildings into destruction and no destruction for an F1-score-optimizing probability threshold, obtained from random sampling, after combining pixel-wise p-values in Mariupol. The Russian invasion of Ukraine started on February 24, 2022. According to reports, the siege of Mariupol intensified after March 2 [Gunter, 2022]. As the image on January 11, 2022 is unfortunately faulty, we had to remove the two 12-day time periods associated with the image from the analysis. The time periods directly before and after are evidently affected by this removal, as pixels generally exhibit lower p-values. Nonetheless, the detection remains mostly stable. The background of each image is an optical Sentinel-2A image (freely available) February 10, 2021 based on bands 4, 3 and 2.

11 Mariupol - ROC & PR Curves

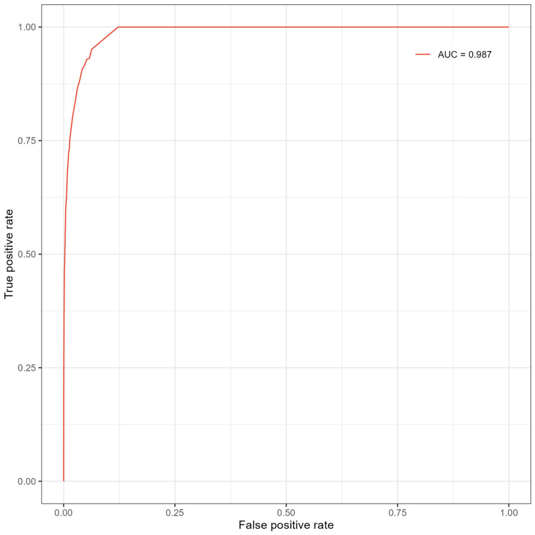

(a) ROC curve

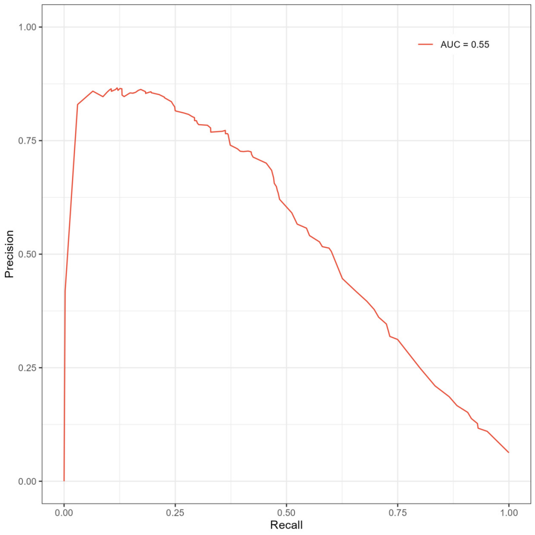

(b) PR curve

Fig. S11: Performance curves of building pixels in Mariupol, Zhovtnevyi district.

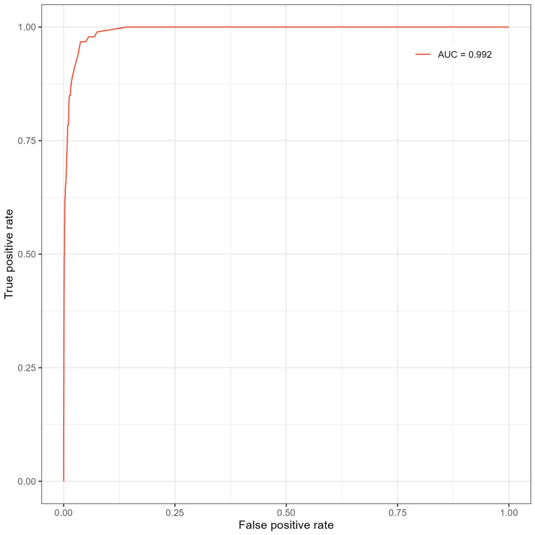

(a) ROC curve

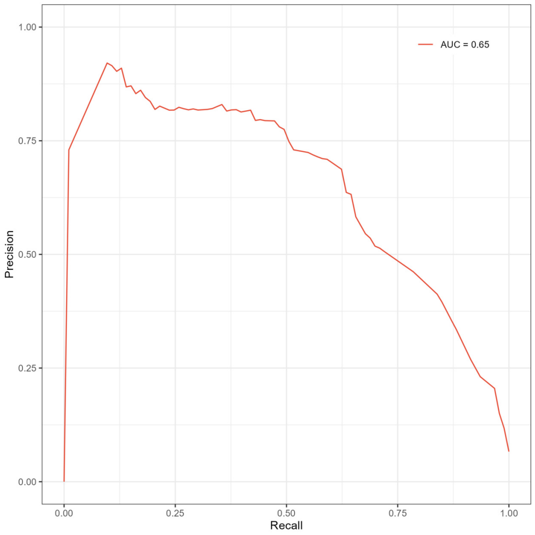

(b) PR curve

Fig. S12: Performance curves of buildings in Mariupol, Zhovtnevyi district.

12 Gaza - P-Values

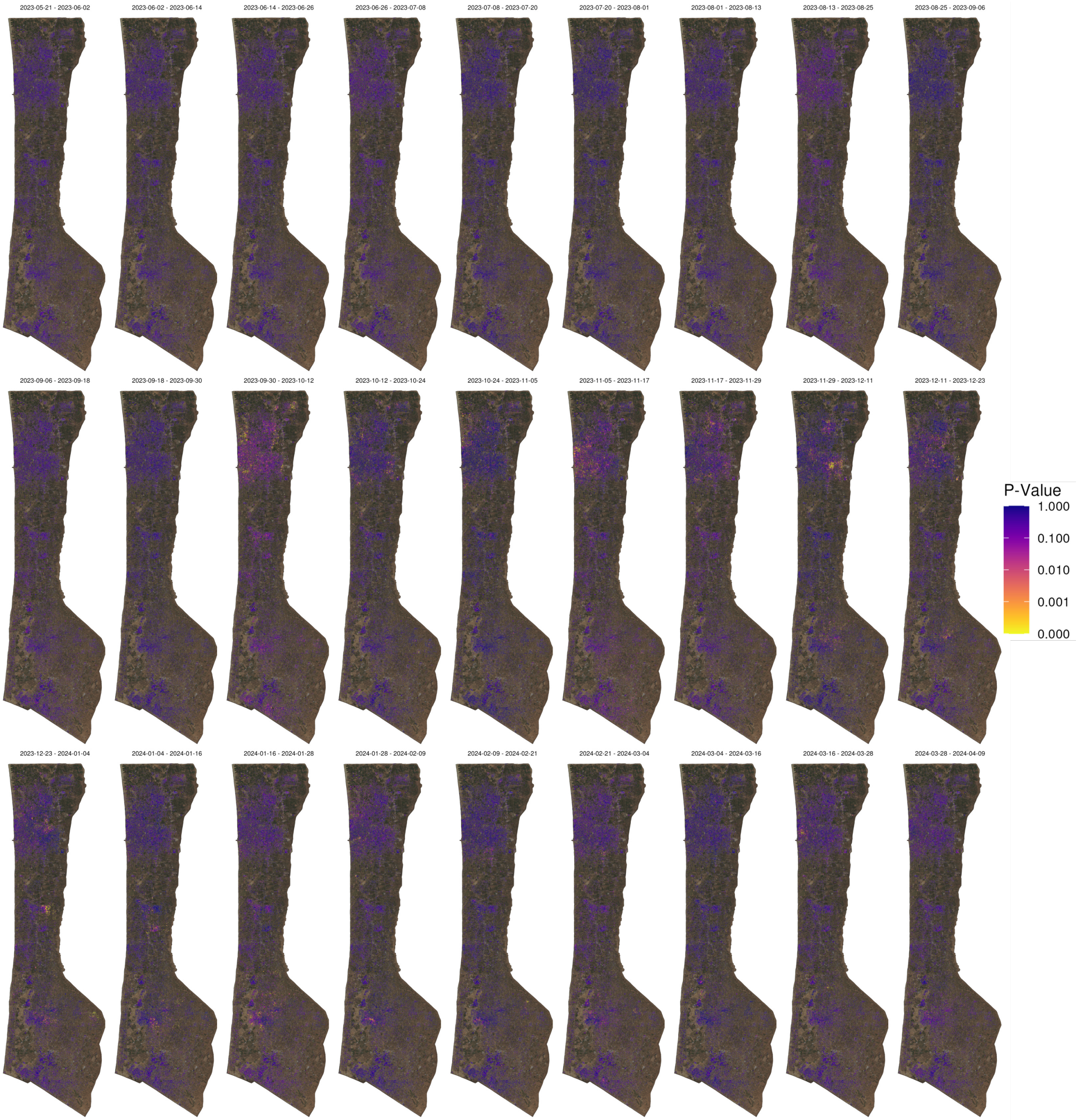

Fig. S13: P-values of destruction of all 10m×10m building pixels in the Gaza Strip over all 12-day time periods. Lower p-values indicate a higher likelihood that part of a building was destroyed. The Israel-Hamas war started on October 7, 2023. The background of each image is an optical Sentinel-2A image (publicly available) from May 5, 2023 based on bands 4, 3 and 2.

13 Gaza - Building Destruction

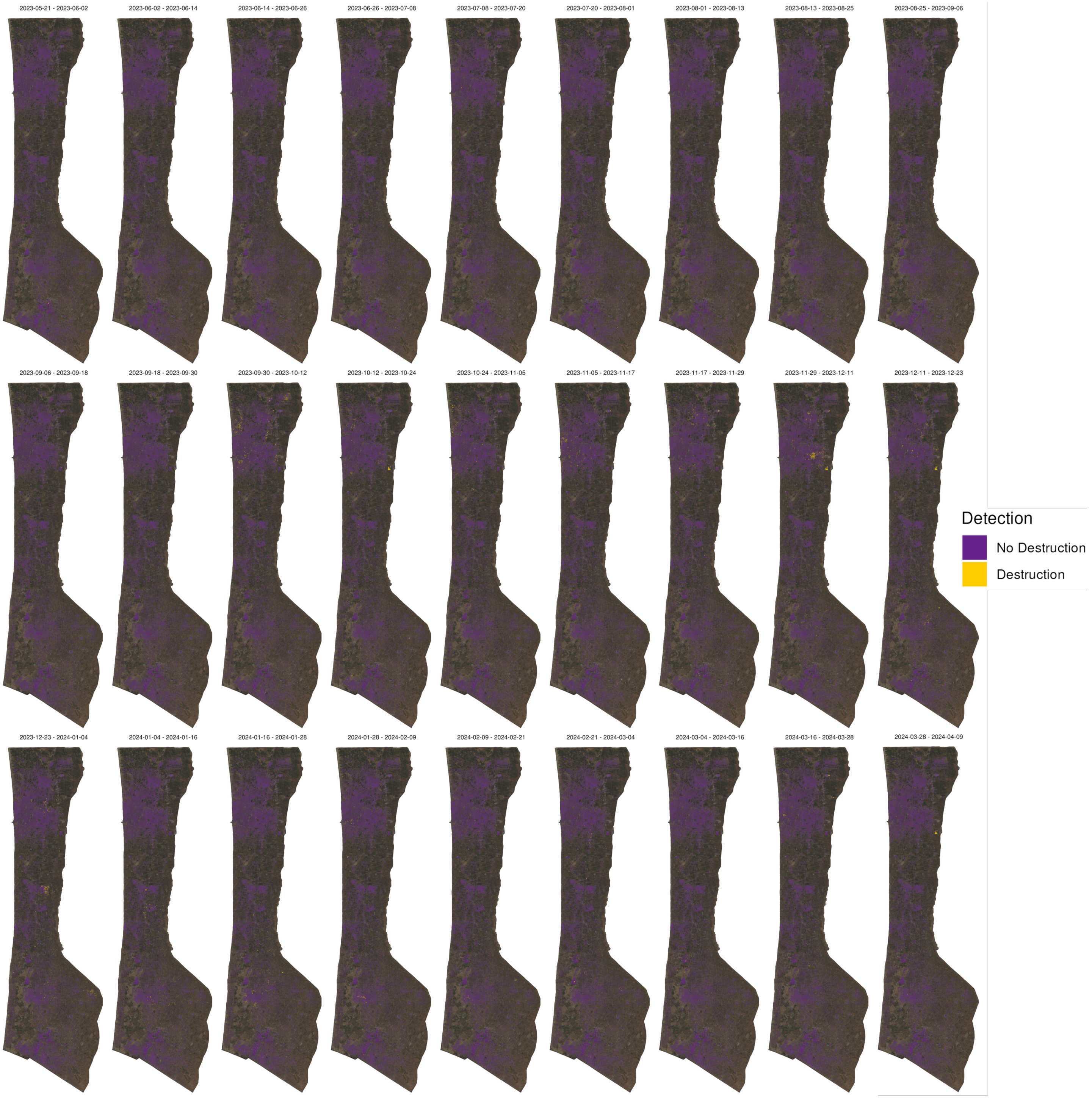

Fig. S14: Classification of entire buildings into destruction and no destruction in Gaza over 12-day time periods for the F1-score-optimizing probability threshold obtained from Mariupol. The Israel-Hamas war started on October 7, 2023. The background of each image is an optical Sentinel-2A image (publicly available) from May 5, 2023 based on bands 4, 3 and 2.

14 Non-parametric Median Regression - Level of Flexibility

As discussed in the main manuscript, we fit a non-parametric median regression on the first differences of the interferometric coherence scores of each pixel over time. For this we employ the R package *quantreg* [Koenker, 2005], which allows for different levels of flexibility (λ) in the fitted trend. A lower λ means a more flexible, i.e., non-linear, trend can be fitted, as visualized in Fig. S15. For $\lambda \to \infty$ the fitted trend approaches linearity. Particularly for longer time series, with potentially multiple break points (e.g., due to destruction and subsequent reconstruction) more flexible fits are likely beneficial compared to simple linear trends.

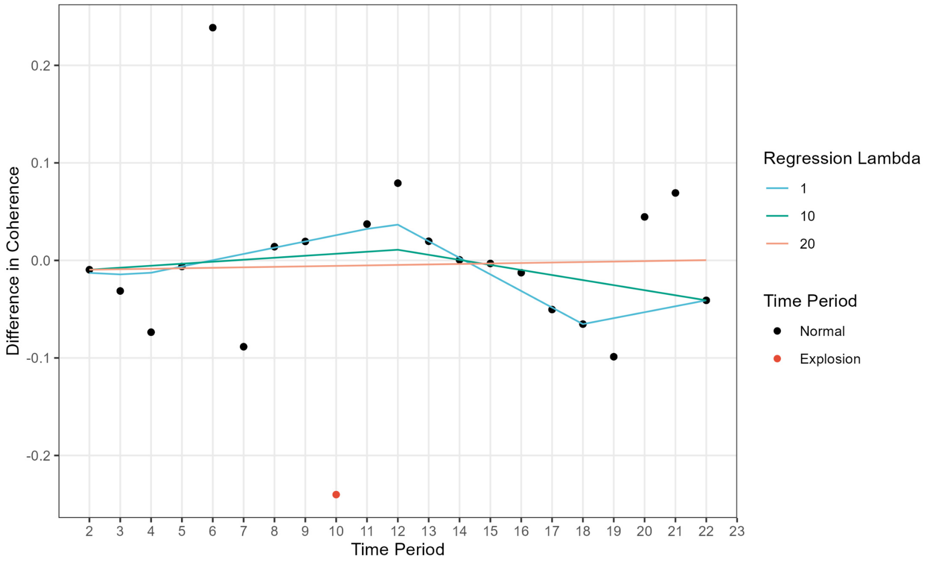

Fig. S15: Median regression for varying levels of flexibility. The points depict the first differences of the coherence scores of a single pixel of a destroyed building in Beirut over time.

In Table S3 and Table S4, we present performance results for varying values of λ for both pixels and buildings. The results indicate that low λ values result in substantially worse performance. Once λ reaches a sufficiently large value, performance stabilizes. Although $\lambda = 10$ demonstrates higher performance in some cases (e.g., F1 score for pixels) and greater stability (e.g., AUPRC standard deviation for buildings), we conclude that a linear trend would generally suffice for this particular use case. Nevertheless, for the reasons outlined previously, we opt for $\lambda = 10$ and thus a medium level of flexibility.

Table S3: Pixel Performance

Flexibility	AUROC	AUPRC	F1
$\lambda = 1$	0.986 (<0.001)	0.568 (0.009)	0.608 (0.005)
$\lambda = 5$	0.989 (<0.001)	0.650 (0.010)	0.663 (0.005)
$\lambda = 7.5$	0.989 (<0.001)	0.651 (0.010)	0.657 (0.004)
$\lambda = 10$	0.989 (<0.001)	0.657 (0.010)	0.663 (0.004)
$\lambda = 12.5$	0.989 (<0.001)	0.657 (0.010)	0.661 (0.004)
$\lambda = 15$	0.989 (<0.001)	0.658 (0.010)	0.661 (0.004)
$\lambda = 20$	0.989 (<0.001)	0.658 (0.010)	0.661 (0.004)

Note: Pixel performance scores in Beirut for varying levels of flexibility (λ) in the non-parametric regression, using random sampling for evaluation. The mean scores across all 100 repetitions (see main manuscript for more info) are reported, with the standard deviation in brackets. The F1 score is reported for the F1-optimizing probability threshold.

Table S4: Building Performance

**Flexibility**	**AUROC**	**AUPRC**	**F1**
$\lambda = 1$	0.997 (<0.001)	0.632 (0.050)	0.744 (0.047)
$\lambda = 5$	0.999 (<0.001)	0.901 (0.056)	0.891 (0.036)
$\lambda = 7.5$	0.999 (<0.001)	0.900 (0.057)	0.891 (0.036)
$\lambda = 10$	0.999 (<0.001)	0.905 (0.058)	0.904 (0.032)
$\lambda = 12.5$	0.999 (<0.001)	0.904 (0.065)	0.907 (0.033)
$\lambda = 15$	0.999 (<0.001)	0.904 (0.064)	0.908 (0.032)
$\lambda = 20$	0.999 (<0.001)	0.905 (0.064)	0.908 (0.032)

Note: Building performance scores in Beirut for varying levels of flexibility (λ) in the non-parametric regression, using random sampling for evaluation. The mean scores across all 100 repetitions (see main manuscript for more info) are reported, with the standard deviation in brackets. The F1 score is reported for the F1-optimizing probability threshold.

15 Distribution of Residuals

For the calculation of p-values, recall that we assume the residuals follow a normal distribution, except in cases where a building has been destroyed. Specifically, $R_{i,t} \sim \text{Normal}(0, \hat{\sigma}_i^2)$, where $\hat{\sigma}_i^2$ represents the standard deviation estimated using the robust scale estimator Q_n.

In Fig. S16, we visualize the distribution of the residuals. The residuals generally appear to follow a normal distribution, though with noticeably larger tails, which we attribute to outliers resulting from the harbor explosion and some additional noise. Due to the differencing applied in our method, outliers occur in both directions. A drop in coherence caused by the destruction of a building is typically followed by an increase in coherence in the subsequent time period as the pixel stabilizes again.

We also observe a more pronounced peak in the residual distribution (see Fig. S16a). To account for this, we considered approximating the empirical distribution using a Laplace distribution instead. Interestingly, this change had minimal impact on the p-values and classification performance, confirming the robustness of our results.

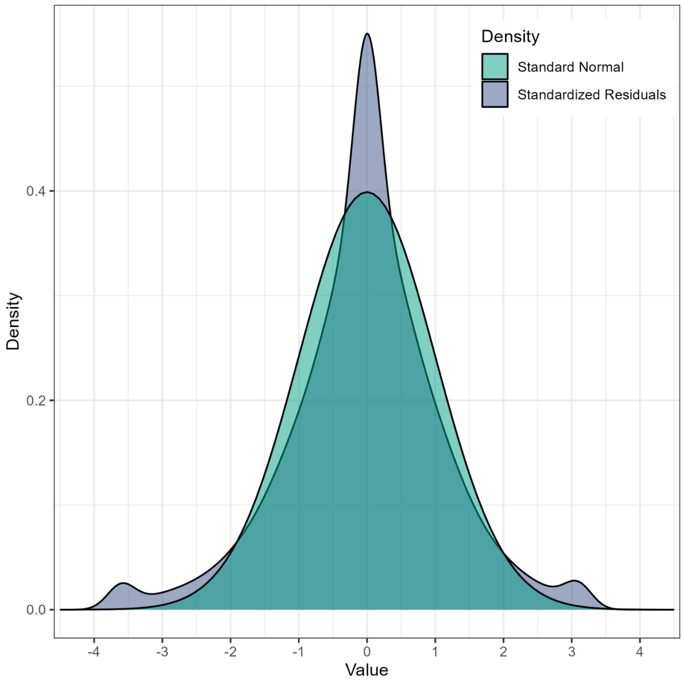

(a) Comparison of standard normal distribution (green) with the empirical distribution of the standardized residuals (blue). The standard deviation of each pixel (multiple residuals) is estimated using the robust estimator Q_n.

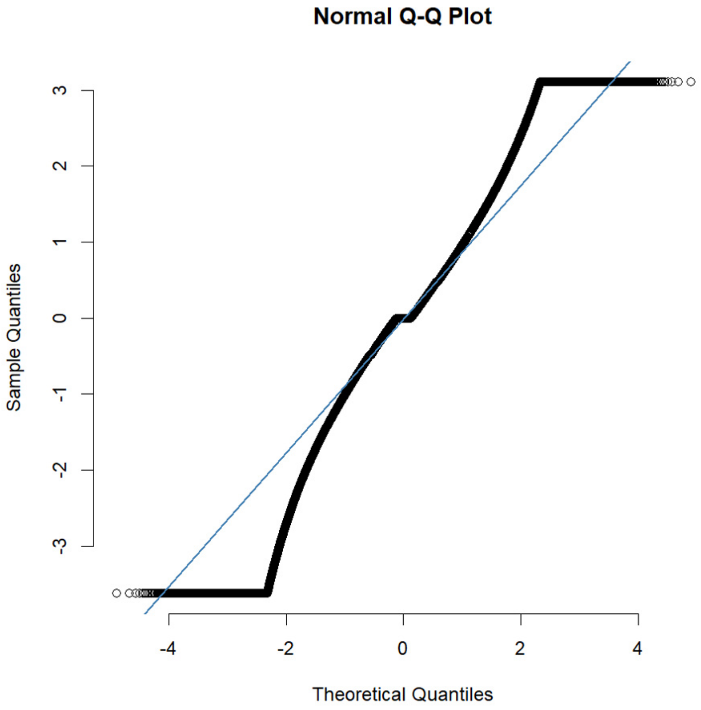

(b) Quantile-quantile (Q-Q) plot of the standardized residuals compared to a standard normal distribution. The standard deviation of each pixel (multiple residuals) is estimated using the robust estimator Q_n.

Fig. S16: Distribution of all building pixel residuals obtained from the median regression ($\lambda = 10$) on the first differences of the coherence scores.

16 Alternative Robust Estimators for Standard Deviation

In the literature, a variety of robust scale estimators are available for estimating the standard deviation of the residuals obtained from the median regression. Here, we consider two widely used alternatives. The most well-known robust scale estimator is the median absolute deviation (MAD) [Lax, 1985], defined as

$$\text{MAD} = b\, med_i\{|x_i - med_j(x_y)|\},$$

which calculates the median deviation from the median over all samples x, and where b is a constant needed for consistency. Another alternative is the S_n estimator, defined as

$$S_n = c\, med_i\{med_j(x_i - x_j)\},$$

where c is a constant needed for consistency. For each i, S_n computes the median of $|x_i - x_j|$ over all $j = 1, .., n$, and then takes the median of these terms over all $i = 1, .., n$. The estimator was introduced by Rousseeuw and Croux [1993], with its origins dating back to Tukey [1977]. Both MAD and S_n offer slightly more robustness than Q_n, but their efficiency for normal distributions is substantially lower [Rousseeuw and Croux, 1993].

We evaluate the performance using both MAD and S_n as alternatives to Q_n in Table S5 and Table S6. While the differences compared to Q_n are rather minor (see Table S3 and Table S4), using Q_n consistently yields better performance scores.

Table S5: Pixel Performance - Alternative Robust SD Estimators

Estimator	Flexibility	AUROC	AUPRC	F1
MAD	$\lambda = 1$	0.985 (<0.001)	0.508 (0.008)	0.561 (0.005)
MAD	$\lambda = 5$	0.988 (<0.001)	0.612 (0.010)	0.634 (0.004)
MAD	$\lambda = 7.5$	0.988 (<0.001)	0.617 (0.009)	0.637 (0.004)
MAD	$\lambda = 10$	0.988 (<0.001)	0.623 (0.009)	0.642 (0.004)
MAD	$\lambda = 12.5$	0.988 (<0.001)	0.622 (0.010)	0.639 (0.005)
MAD	$\lambda = 15$	0.988 (<0.001)	0.622 (0.009)	0.640 (0.005)
MAD	$\lambda = 20$	0.988 (<0.001)	0.622 (0.009)	0.640 (0.004)
S_n	$\lambda = 1$	0.986 (<0.001)	0.541 (0.008)	0.591 (0.005)
S_n	$\lambda = 5$	0.988 (<0.001)	0.634 (0.010)	0.649 (0.004)
S_n	$\lambda = 7.5$	0.989 (<0.001)	0.635 (0.010)	0.649 (0.004)
S_n	$\lambda = 10$	0.989 (<0.001)	0.641 (0.010)	0.653 (0.004)
S_n	$\lambda = 12.5$	0.989 (<0.001)	0.642 (0.010)	0.654 (0.004)
S_n	$\lambda = 15$	0.989 (<0.001)	0.642 (0.009)	0.654 (0.004)
S_n	$\lambda = 20$	0.989 (<0.001)	0.642 (0.009)	0.654 (0.004)

Note: Pixel performance scores in Beirut for varying levels of flexibility (λ) in the non-parametric regression, using both MAD and S_n instead of Q_n for estimating the standard deviation of the residuals. Uses random sampling for evaluation. The mean scores across all 100 repetitions (see main manuscript for more info) are reported, with the standard deviation in brackets. The F1 score is reported for the F1-optimizing probability threshold.

Table S6: Building Performance - Alternative Robust SD Estimators

Estimator	Flexibility	AUROC	AUPRC	F1
S_n	$\lambda = 1$	0.997 (<0.001)	0.570 (0.052)	0.740 (0.042)
S_n	$\lambda = 5$	0.999 (<0.001)	0.897 (0.058)	0.881 (0.027)
S_n	$\lambda = 7.5$	0.999 (<0.001)	0.892 (0.060)	0.866 (0.040)
S_n	$\lambda = 10$	0.999 (<0.001)	0.896 (0.062)	0.880 (0.037)
S_n	$\lambda = 12.5$	0.999 (<0.001)	0.894 (0.068)	0.884 (0.037)
S_n	$\lambda = 15$	0.999 (<0.001)	0.897 (0.068)	0.886 (0.036)
S_n	$\lambda = 20$	0.999 (<0.001)	0.898 (0.066)	0.887 (0.036)
MAD	$\lambda = 1$	0.997 (<0.001)	0.550 (0.052)	0.726 (0.051)
MAD	$\lambda = 5$	0.999 (<0.001)	0.873 (0.056)	0.867 (0.039)
MAD	$\lambda = 7.5$	0.999 (<0.001)	0.877 (0.055)	0.871 (0.037)
MAD	$\lambda = 10$	0.999 (<0.001)	0.880 (0.055)	0.887 (0.042)
MAD	$\lambda = 12.5$	0.999 (<0.001)	0.900 (0.065)	0.898 (0.039)
MAD	$\lambda = 15$	0.999 (<0.001)	0.901 (0.063)	0.900 (0.039)
MAD	$\lambda = 20$	0.999 (<0.001)	0.901 (0.064)	0.898 (0.040)

Note: Building performance scores in Beirut for varying levels of flexibility (λ) in the non-parametric regression, using both MAD and S_n instead of Q_n for estimating the standard deviation of the residuals. Uses random sampling for evaluation. The mean scores across all 100 repetitions (see main manuscript for more info) are reported, with the standard deviation in brackets. The F1 score is reported for the F1-optimizing probability threshold.

17 Interferometric Coherence Scores - Using Absolute Values

As outlined in the Methods section of the main manuscript, we first calculate the first differences of the interferometric coherence scores for each pixel over time, before fitting the median regression. This step improves performance, likely because the changes in coherence become more pronounced when focusing on differences rather than absolute values.

In Table S7 and Table S8, we report performance scores when the median regression is fitted directly to the coherence scores, without taking the first differences. The results are presented for varying levels of flexibility (λ) for both pixels and buildings. As shown, performance is substantially worse compared to our preferred approach. Interestingly, in this alternative setup, a more flexible fit (lower λ) appears to be beneficial, suggesting that a higher flexibility compensates, to some extent, for the lack of differencing.

Table S7: Pixel Performance

Flexibility	AUROC	AUPRC	F1
$\lambda = 0.1$	0.880 (<0.001)	0.168 (0.004)	0.268 (0.003)
$\lambda = 1$	0.972 (<0.001)	0.360 (0.006)	0.455 (0.004)
$\lambda = 5$	0.976 (<0.001)	0.340 (0.006)	0.437 (0.004)
$\lambda = 7.5$	0.974 (<0.001)	0.313 (0.005)	0.405 (0.004)
$\lambda = 10$	0.974 (<0.001)	0.308 (0.005)	0.401 (0.004)
$\lambda = 12.5$	0.974 (<0.001)	0.313 (0.005)	0.403 (0.004)
$\lambda = 15$	0.976 (<0.001)	0.325 (0.005)	0.415 (0.004)
$\lambda = 20$	0.977 (<0.001)	0.333 (0.005)	0.416 (0.004)

Note: Pixel performance scores in Beirut for varying levels of flexibility (λ) in the non-parametric regression, **without** taking the first of the coherence scores, using random sampling for evaluation. The mean scores across all 100 repetitions (see main manuscript for more info) are reported, with the standard deviation in brackets. The F1 score is reported for the F1-optimizing probability threshold.

Table S8: Building Performance

Flexibility	AUROC	AUPRC	F1
$\lambda = 0.1$	0.976 (0.001)	0.514 (0.052)	0.653 (0.039)
$\lambda = 1$	0.996 (<0.001)	0.519 (0.060)	0.703 (0.043)
$\lambda = 5$	0.996 (<0.001)	0.673 (0.088)	0.696 (0.041)
$\lambda = 7.5$	0.996 (<0.001)	0.692 (0.087)	0.710 (0.047)
$\lambda = 10$	0.996 (<0.001)	0.678 (0.086)	0.703 (0.045)
$\lambda = 12.5$	0.996 (<0.001)	0.663 (0.084)	0.677 (0.038)
$\lambda = 15$	0.996 (<0.001)	0.660 (0.083)	0.669 (0.046)
$\lambda = 20$	0.996 (<0.001)	0.667 (0.085)	0.684 (0.048)

Note: Building performance scores in Beirut for varying levels of flexibility (λ) in the non-parametric regression, **without** taking the first of the coherence scores, using random sampling for evaluation. The mean scores across all 100 repetitions (see main manuscript for more info) are reported, with the standard deviation in brackets. The F1 score is reported for the F1-optimizing probability threshold.

18 Destruction Labels in Gaza

As discussed in the main manuscript, we conduct only a composite damage analysis for Gaza, despite the availability of destruction labels from UNOSAT. In practice, we find slight imprecisions in both the UNOSAT labels and the OSM building footprints, when compared to the actual locations of buildings in Google Earth. Together, these discrepancies create difficulties in reliably matching the label coordinates to the OSM building polygons, which would be necessary for an individual performance analysis.

We illustrate these challenges in Fig. S17, which provides examples directly taken from QGIS. Even with a nearest neighbor matching approach and a maximum distance threshold, many label coordinates (red points) cannot be accurately matched to the corresponding OSM building footprints (green polygons). Hence, any individual performance evaluation would risk introducing biases. Thus, instead, we opted to perform a composite damage analysis for Gaza.

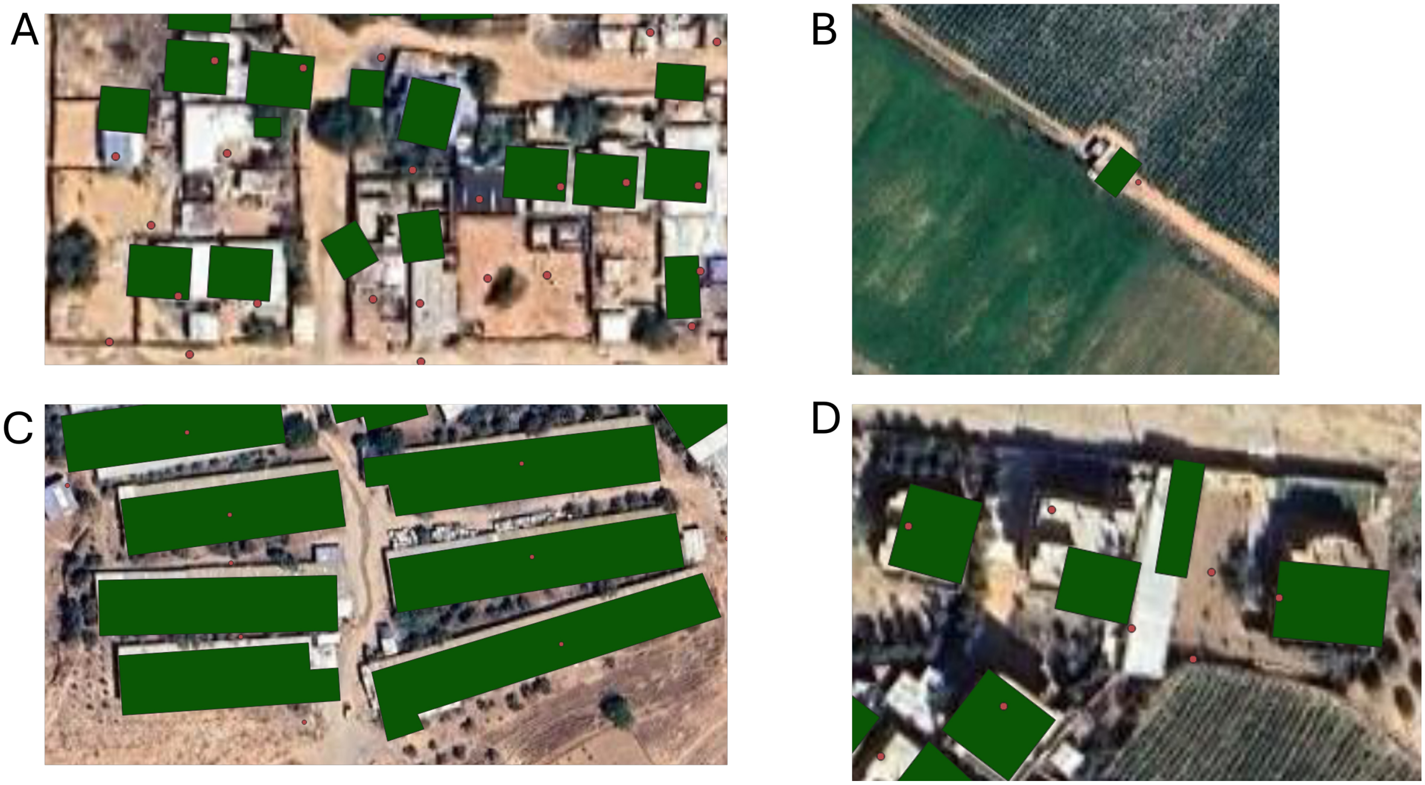

Fig. S17: Examples for matching UNOSAT labels to buildings in Gaza. The red points denote the coordinates of destruction labels provided by UNOSAT. The green polygons represent OSM building footprints. The background of each example is the most current satellite image provided by Google Earth.

19 Spatial Validation Analysis for Gaza

Due to the discrepancies between UNOSAT labels and OSM building footprints in Gaza (see Supp.18), we do not conduct an individual performance analysis, and instead only opt for a composite damage analysis in our main manuscript. Here, we additionally report performance results from a spatial validation analysis.

Specifically, we take the UNOSAT destruction labels as reference points and draw circles of varying radii around them. For each circle, we then check whether it contains at least one building classified as destroyed by our algorithm. We report accuracy scores for all available dates in our analysis period in Table S9. As expected, the 10m scores are relatively low, reflecting the misalignment between UNOSAT annotations and OSM building footprints. At 50m, however, on average about half of the UNOSAT labels contain a building marked as destroyed in their radius, with further increases at 100m. Importantly, in the time periods before the war, only 0.021% of all buildings are (incorrectly) classified as destroyed, hence the false-positive rate is low. Overall, these findings provide further evidence that the algorithm can reliably detect patterns of building destruction.

Table S9: Accuracy scores for varying distances

Date	10m Accuracy	50m Accuracy	100m Accuracy
Oct. 10, 2023	0.291	0.464	0.552
Nov. 11, 2023	0.204	0.393	0.536
Nov. 26, 2023	0.241	0.472	0.658
Jan. 7, 2024	0.265	0.511	0.707
Feb. 29, 2024	0.280	0.559	0.772
Apr 1, 2024	0.277	0.556	0.774

Note: Accuracy scores of destruction detection for varying distances. We use the optimal probability threshold from the Mariupol case study to classify buildings as destroyed. For each date, we derive circles with radii of 10m, 50m, and 100m around each UNOSAT destruction label. If a circle contains at least one building marked as destroyed by our algorithm, the corresponding UNOSAT label is counted as correctly identified. We repeat this for all labels and report the overall share of correctly identified buildings for each radius. Note, since image acquisition dates and labeling dates do not perfectly align, we use the closest possible match (see Table 2 in the main manuscript). This results in substantially lower scores for Nov. 11, as our classification is conducted using images from Nov. 6. A radius of 10m covers roughly 1.8 buildings, a radius of 50m covers 12 buildings, and a radius of 100m covers 36.2 buildings.

References

Joel Gunter. Ukrainian city of mariupol 'near to humanitarian catastrophe' after bombardment, 2022. URL `https://www.bbc.com/news/world-europe-60585603`. https://www.bbc.com/news/world-europe-60585603, Retrieved 2024-11-21.

Roger Koenker. Quantile regression. *Cambridge University Press*, 2005.

David A Lax. Robust estimators of scale: Finite-sample performance in long-tailed symmetric distributions. *Journal of the American Statistical Association*, 80(391):736–741, 1985.

Peter J Rousseeuw and Christophe Croux. Alternatives to the median absolute deviation. *Journal of the American Statistical association*, 88(424):1273–1283, 1993.

John W Tukey. Exploratory data analysis. *Reading/Addison-Wesley*, 1977.
